# Supplementary material for: Revisiting the Zingiberales: using multiplexed exon capture to resolve ancient and recent phylogenetic splits in a charismatic plant lineage
Source: PeerJ. 2016 Jan 21;4:e1584. doi: 10.7717/peerj.1584 (PMC4727956; doi:10.7717/peerj.1584)
Supplement: Supplemental Information 1 [file peerj-04-1584-s001.docx]

**Supplementary Methods**

This document outlines the bioinformatic processing steps that were used. When appropriate, the perl scripts and associated files are referenced. In other cases, command syntax for external software, a bash command file, or a description of a process is given. The commands and command files (perl or bash) are given a fixed width font and highlighted in the steps where they are executed, for clarity. The commands used for generating the data are given, although some faster and more memory efficient methods have more recently become available. In any future utilizations of the pipeline, more efficient updated versions of the pipeline should likely be used and will be available at https://github.com/chodon/zingiberales.

1. **Pre-process transcriptomes:** Transcriptomes were often sequenced before Illumina converted to Illumina 1.8+ quality scores. Because most external software use Illumina 1.8+ quality encoding by default, quality scores were converted prior to any other processing. Low complexity, low quality, adapter sequences, and PCR duplicates were removed and overlapping paired-end reads were merged.
2. Convert quality scores: converter.pl
3. Clean and merge reads: sonal_2scrubReads_cs2.pl
4. **Pre-process *Musa* *acuminata* CDS:** The gene annotation file accompanying the draft *Musa acuminata* genome was used to generate a .bed format file that contained the location of the exon boundaries in the draft genome coding DNA sequences file (CDS file). This .bed format file was then used to split each gene into its component exons.
5. extract separate exons: parse_gff_to_bed.pl
6. **Align, SNP call, genotype, and print for tiling for Agilent array:** Each cleaned transcriptome was aligned to the *Musa acuminata* CDS which had been separated into exons using novoalign with very lenient alignment stringency to enable reads with many substitutions to align. SNPs were called with SAMtools and VarScan. Retained exons had a length of at least 150 bp and had coverage in each of the 7 additional families. The SNP calls were used to print out a family-specific bait, after adjusting for insertions and deletions. The list of genes were culled to remove genes with unusually high or low GC content, that blasted to self, or were matched by the repeatMasker database. Finally, an external software was used to divide the exons into 60mer oligos at a specified tiling density (in our case, we used 1x tiling density) for designing the microarray chip to be printed by Agilent.
7. Alignment and SNP calling:
   1. run_8_2_novoalign_full_cds_sepexons.pl
   2. make_pileups.sh

- 1. run_varscan.sh

1. Filter gene list:
   1. Generate list of exons with coverage, length greater than 150bp, make_covered_bed_files.sh

- 1. intersectBedFiles.sh

1. Genotype transcriptome and generate bed files to extract covered portions of exons
   1. Use the SNP calls and the reference sequences to generate a genotype for each transcriptome species: parseVarScanv5.pl
   2. adjust for indels: remove_Zs_and_delete.sh
   3. Gather indel list from printed output of parseVarScanv5.pl. Use indel list as input for editBedv5e.pl to adjust for indels in output from fasta printed out from parseVarScanv5.pl and remove_Zs_and_delete.sh
   4. Extend the length of the exon sequence on an individual basis, even if not covered by all individuals (to help reduce edge effects): add_slops.pl
2. Filtering gene list part 2
   1. Remove genes that have 30>GC%>70: countGC.pl
   2. Remove genes which blast to self: blastn -db musa.slopped.fasta -query musa.slopped.fasta -outfmt 6 | awk '$1!=$2' | awk '{print $1}' | sort | uniq
   3. Remove repeatMasker hits
3. Generate array: use array_design.pl (author Hernan A. Burbano, MPI-EVA and Kay Pruefer svn co http://biofs04/svn/bioinf/motiv-counts/) to design tiling for printing by Agilent.
4. **Pre-process captured reads:** These steps are outlined in Figure 1b. In brief, raw reads are cleaned and merged, individual references are created by an iterative SNP calling process to the bait reference exons, these references are extended with Mapsembler, final SNP calls are made and genotypes are made by parsing and filtering the SNP call file.
   1. 1PreCleanup.pl (https://github.com/MVZSEQ/denovoTargetCapturePhylogenomics)
   2. 2ScrubReads.pl (https://github.com/MVZSEQ/denovoTargetCapturePhylogenomics)
   3. perl uZingNucAlignFix_140923_famx.pl –Z famx.final.fasta
   4. postAlignFixMapsemble.pl –L LIBX –Z anything (this script depends on 7recipBlasting.pl (written by Sonal Singhal as part of Singhal 2013).
   5. uZingSNPCall_150128.pl
   6. uZingGATK2Fa_150129.pl
5. **Align genotyped fasta files, filter genes**
   1. grepForAllFromConcatenatedFastaC.sh
   2. mafft --maxiterate 1000 --localpair --thread 2 GENE.fasta > GENE.aligned.fasta
   3. Find bait edges in alignment: mothur>summary.seqs(fasta=GENE.aligned.fasta)
   4. Trim to MUAC boundaries: selectsites.pl –x 1 –s [muac boundaries from mothur output] GENE.aligned.fasta > GENE.B.fas (selectsites.pl can be found here:http://raven.iab.alaska.edu/~ntakebay/teaching/programming/perl-scripts/perl-scripts.html)
   5. Trim to coding position starting with 1^st^ position utilizing position from *Musa* cds
   6. Run macse to place sequences into coding frame:

java -Xmx12g -jar macse_v1.01b.jar -prog alignSequences -seq GENE.aligned.trimmed2.fas

- 1. check for internal frameshifts README_frameshiftCheck.txt
  2. align by codon position with prank

prank -d=geneIn -o=geneOut -codon -gaprate=0.009 -gapext=0.5 -iterate=20

- 1. run RAxML to test gene tree length

raxmlHPC-SSE3 -m GTRGAMMA -p 12345 -N 10 -s geneOut -n raxOut

- 1. remove genes with skewed tree length

1. **Phylogenetic analyses**
   1. PartitionFinder: see attached .cfg and starting tree files
   2. Maximum likelihood search and bootstrapping: raxmlHPC-HYBRID -T 4 -n result -s infile.txt -q part.txt -p 12345 -x 12345 -N 1000 -c 25 -f a -m GTRCAT [see attached infile and part.txt file]
   3. Parsimony search: paup> HSearch addSeq=random nreps=100 rseed=725638180
   4. Parsimony bootstrap: paup> Bootstrap nreps=1000 seed=1486916772 / nreps=10
   5. Maximum likelihood gene trees and gene tree bootstrapping: runRaxml1000boot.pl
   6. ASTRAL-II: java -Xmx8000M -jar astral.4.7.8.jar -i best309trees.txt -b bs_paths -r 1000
