# Supplementary material for: Revisiting the Zingiberales: using multiplexed exon capture to resolve ancient and recent phylogenetic splits in a charismatic plant lineage
Source: PeerJ. 2016 Jan 21;4:e1584. doi: 10.7717/peerj.1584 (PMC4727956; doi:10.7717/peerj.1584)
Supplement: Supplemental Information 2 — The material comes from herbaria, living collections, and cultivated plants. In the case of herbarium material we list the collection number and the herbarium using the standard code (Thiers, continuously updated). In the other cases we list accession numbers and the name of the living collection location. Minimum and median divergence measured by percent identity to the family specific bait is also listed. [file peerj-04-1584-s002.docx]

| **Family** | **Species** | **Voucher** | **Code** | **% ID to Bait**  **minimum \|\| median** |
| --- | --- | --- | --- | --- |
| **Samples sequenced *de novo*** | | | | |
| Cannaceae | *Canna indica* L. | 80856 (Huntington Botanical Garden) | CS37 | 90.60 \|\| 96.20 |
| Cannaceae | *Canna iridiflora* Ruiz & Pav. | 90142 (Huntington Botanical Garden) | CS28 | 83.94 \|\| 96.09 |
| Cannaceae | *Canna jaegeriana* Urb. | M.Bartlett mb0854 (Lyon Arboretum) | CS48 | 90.27 \|\| 95.91 |
| Costaceae | *Chamaecostus acaulis* (S.Moore) T.André & C.D.Specht | 25510 (HUFU) | CS72 | 86.92 \|\| 95.30 |
| Costaceae | *Cheilocostus speciosus* (J.Koenig) C.D.Specht | L-67.1091 (Lyon Arboretum) | CS15 | 83.72 \|\| 96.01 |
| Costaceae | *Costus dubius* (Afzel.) K.Schum. | 89-0918 (U. California Botanical Garden) | CS42 | 88.02 \|\| 97.11 |
| Costaceae | *Costus gabonensis* Koechlin | PJM Maas M10291 | CS49 | 87.73 \|\| 97.22 |
| Costaceae | *Costus osae* Maas & H.Maas | 89894*1 (Huntington Botanical Garden) | CS52 | 88.62 \|\| 96.71 |
| Costaceae | *Costus pictus* D.Don | 94-685 GH (United States Botanical Research Greenhouse (Smithsonian Institution)) | CS44 | 88.62 \|\| 96.70 |
| Costaceae | *Costus pulverulentus* C.Presl | 2004-0330A (Fairchild Tropical Botanical Garden) | CS45 | 88.62 \|\| 96.66 |
| Costaceae | *Dimerocostus strobilaceus* Kuntze | 2001-0595B (Fairchild Tropical Botanical Garden) | CS17 | 80.43 \|\| 95.24 |
| Costaceae | *Monocostus uniflorus* (Poepp. ex Petersen) Maas | 2000 894H (Fairchild Tropical Botanical Garden) | CS19 | 87.10 \|\| 95.13 |
| Costaceae | *Tapeinochilos ananassae* (Hassk.) K.Schum. | 83458B (Fairchild Tropical Botanical Garden) | CS27 | 87.18 \|\| 95.97 |
| Heliconiaceae | *Heliconia acuminata* A.Rich. | USBRG 1994-727 (United States Botanical Research Greenhouse (Smithsonian Institution)) | CS34 | 89.94 \|\| 98.36 |
| Heliconiaceae | *Heliconia nutans* Woodson | 3311403 (US) | CS69 | 90.32 \|\| 98.00 |
| Heliconiaceae | *Heliconia* sp. 23 | 58523A (Fairchild Tropical Botanical Garden) | CS23 | 91.40 \|\| 98.25 |
| Heliconiaceae | *Heliconia* sp. 25 | 10823 (HNT) | CS25 | 73.30 \|\| 98.15 |
| Heliconiaceae | *Heliconia* sp. 41 | 90.1609 (U. California Botanical Garden) | CS41 | 89.36 \|\| 98.16 |
| Lowiaceae | *Orchidantha fimbriata* Holttum | 2003-1178A (Fairchild Tropical Botanical Garden) | CS30 | 91.25 \|\| 98.15 |
| Lowiaceae | *Orchidantha maxillarioides* (Ridl.) K.Schum. | 1639/91 (New York Botanical Garden) | CS57 | 92.27 \|\| 98.15 |
| Marantaceae | *Calathea roseopicta* (Linden) Regel | B.AA.033 (U.C. Davis Botanical Conservatory) | CS06 | 87.92 \|\| 95.42 |
| Marantaceae | *Donax canniformis* K.Schum. | L-83.0894 (Lyon Arboretum) | CS50 | 83.11 \|\| 95.38 |
| Marantaceae | *Halopegia azurea* (K.Schum.) K.Schum. | 2003.0185 (Lyon Arboretum) | CS39 | 88.02 \|\| 95.50 |
| Marantaceae | *Ischnosiphon heleniae* L.Andersson | L-86.0493 (Lyon Arboretum) | CS53 | 82.80 \|\| 95.21 |
| Marantaceae | *Marantochloa leucantha* (K.Schum.) Milne-Redh. | L-80.0376 (Lyon Arboretum) | CS24 | 87.08 \|\| 95.53 |
| Marantaceae | *Stromanthe stromanthoides* (J.F.Macbr.) L. Andersson | 93679A (Fairchild Tropical Botanical Garden) | CS18 | 90.83 \|\| 97.35 |
| Marantaceae | *Thaumatococcus daniellii* (Benn.) Benth. | 83374A (Fairchild Tropical Botanical Garden) | CS38 | 88.93 \|\| 95.36 |
| Musaceae | *Ensete superbum* (Roxb.) Cheesman | USBRG 1998-164 (United States Botanical Research Greenhouse (Smithsonian Institution)) | CS51 | 88.12 \|\| 98.03 |
| Musaceae | *Ensete ventricosum* (Welw.) Cheesman | C. Barrett 359 CA (CSLA) | CS14 | 89.56 \|\| 98.05 |
| Musaceae | *Musa basjoo* Siebold & Zucc. ex Iinuma | 89.0873 (U. California Botanical Garden) | CS70 | 86.53 \|\| 98.80 |
| Musaceae | *Musa coccinea* Andrews | ITC0287 (Bioversity) | CS54 | 88.76 \|\| 98.31 |
| Musaceae | *Musa ornata* Roxb. | ITC0637 (Bioversity) | CS55 | 93.56 \|\| 98.80 |
| Musaceae | *Musa* sp. | 2007-0825A (Fairchild Tropical Botanical Garden) | CS12 | 87.80 \|\| 98.86 |
| Musaceae | *Musella lasiocarpa* (Franch.) C.Y.Wu ex H.W.Li | 98 1187A (Fairchild Tropical Botanical Garden) | CS13 | 89.90 \|\| 98.14 |
| Strelitziaceae | *Ravenala madagascariensis* Sonn. | P.1396G (Fairchild Tropical Botanical Garden) | CS35 | 92.86 \|\| 98.22 |
| Strelitziaceae | *Strelitzia caudata* R.A.Dyer | 98443 (Huntington Botanical Garden) | CS43 | 92.62 \|\| 98.03 |
| Strelitziaceae | *Strelitzia reginae* Banks | M. Bartlett mb0607 (ex hort.: Spruce St, Berkeley CA) | CS71 | 93.09 \|\| 96.97 |
| Zingiberaceae | *Aframomum angustifolium* (Sonn.) K.Schum. | 9577A (Fairchild Tropical Botanical Garden) | CS40 | 81.22 \|\| 95.79 |
| Zingiberaceae | *Alpinia purpurata* (Vieill.) K.Schum. | Poulsen 2467 (BSIP,E,AAU) | CS46 | 85.93 \|\| 96.10 |
| Zingiberaceae | *Alpinia zerumbet* (Pers.) B.L.Burtt & R.M.Sm. | X.17-31A (Fairchild Tropical Botanical Garden) | CS29 | 85.96 \|\| 96.00 |
| Zingiberaceae | *Curcuma longa* L. | B.97.097 (U.C. Davis Botanical Conservatory) | CS07 | 86.13 \|\| 96.39 |
| Zingiberaceae | *Curcuma roscoeana* Wall. | 96 1594A (Fairchild Tropical Botanical Garden) | CS47 | 89.36 \|\| 96.51 |
| Zingiberaceae | *Elettariopsis stenosiphon* (K.Schum.) B.L.Burtt & R.M.Sm. | 2001-0839A (Fairchild Tropical Botanical Garden) | CS22 | 74.03 \|\| 96.12 |
| Zingiberaceae | *Etlingera elatior* (Jack) R.M.Sm. | 97-686A (Fairchild Tropical Botanical Garden) | CS36 | 81.31 \|\| 95.94 |
| Zingiberaceae | *Globba winitii* C.H.Wright | B.94.376 (U.C. Davis Botanical Conservatory) | CS09 | 86.11 \|\| 96.17 |
| Zingiberaceae | *Hedychium coronarium* J.Koenig | B.2004.173 (U.C. Davis Botanical Conservatory) | CS02 | 90.27 \|\| 97.04 |
| Zingiberaceae | *Renealmia alpinia* (Rottb.) Maas | 93-740D (Fairchild Tropical Botanical Garden) | CS21 | 88.82 \|\| 96.00 |
| Zingiberaceae | *Riedelia* sp. | 2001-0838A (Fairchild Tropical Botanical Garden) | CS16 | 86.84 \|\| 96.30 |
| Zingiberaceae | *Scaphochlamys* sp. | L-83.0941 (Lyon Arboretum) | CS33 | 86.73 \|\| 97.03 |
| Zingiberaceae | *Siamanthus siliquosus* K.Larsen & J.Mood | 1995-187 (United States Botanical Research Greenhouse (Smithsonian Institution)) | CS58 | 82.25 \|\| 96.41 |
| Zingiberaceae | *Siphonochilus kirkii* (Hook.f.) B.L.Burtt | 2004-0324A (Fairchild Tropical Botanical Garden) | CS32 | 74.75 \|\| 94.37 |
| Zingiberaceae | *Zingiber officinale* Roscoe | UCBG 90-1128 (U. California Botanical Garden) | CS20 | 87.90 \|\| 96.95 |
| Zingiberaceae | *Zingiber spectabile* Griff. | 95-825A (Fairchild Tropical Botanical Garden) | CS31 | 86.75 \|\| 97.45 |
| **Genomic resources used in phylogeny** | | | | |
| Arecaceae | *Sabal bermudana* L.H.Bailey | Transcriptome data (OneKP, acc. HWUP) | HWUP |  |
| Hanguanaceae | *Hanguana malayana* Merr. | Transcriptome data (MonAToL, acc. Hanguana malayana) | HAMA |  |
| Typhaceae | *Typha angustifolia* L. | Transcriptome data (OneKP, acc. PPQR) | PPQR |  |
| Musaceae | *Musa acuminata* Colla subsp. *malaccencis* (Ridl.) Nasution DH-Pahang v1 | Whole genome data, GI 400259569 | MUAC |  |
| Musaceae | *Musa balbisiana* Colla “Pisung Klutuk Wulung” | Whole genome data, SAMN02333823 | MUBA |  |
| **Genomic resources used to generate bait** | | | | |
| Costaceae | *Costus pulverulentus* C.Presl | Transcriptome data (MonAToL, acc. *Costus pulverulentus)* |  | 86.18 \|\| 92.42 |
| Zingiberaceae | *Zingiber officinale* Roscoe | Transcriptome data (1KP, acc. BDJQ) |  | 87.50 \|\| 92.31 |
| Zingiberaceae | *Curcuma longa* L. | Transcriptome data (1KP, acc. JQCX) |  |  |
| Zingiberaceae | *Curcuma longa* L. | Transcriptome data (1KP, acc. OYLU) |  |  |
| Lowiaceae | *Orchidantha maxillarioides* K.Schum. | Transcriptome data (1KP, acc. LSKK) |  | 88.59 \|\| 94.08 |
| Cannaceae | *Canna* sp*.* | Transcriptome data (1KP, acc. TZNS) |  | 86.44 \|\| 92.93 |
| Strelitziaceae | *Strelitzia reginae* Banks | Transcriptome data (1KP, acc. UOEL) |  | 90.24 \|\| 95.16 |
| Marantaceae | *Maranta leuconeura* E.Morren | Transcriptome data (1KP, acc. JNUB) |  | 88.75 \|\| 92.68 |
| Heliconiaceae | *Heliconia* sp. | Transcriptome data (1KP, acc. KNKV) |  | 89.32 \|\| 94.79 |
| Musaceae | *Musa acuminata* Colla subsp. *malaccencis* (Ridl.) Nasution DH-Pahang v1 | Whole genome data, GI 400259569 |  | |
| Costaceae | *Costus pulverulentus* C.Presl | GI 557637393 |  |  |
| Zingiberaceae | *Zingiber spectabile* Griff. | GI 456061535 |  |  |
| Heliconiaceae | *Heliconia* sp. | GI 456061430 |  |  |
| Zingiberaceae | *Alpinia zerumbet* (Pers.) B.L.Burtt & R.M.Sm. | GI 449326771 |  |  |
| Lowiaceae | *Orchidantha fimbriata* Holttum | GI 557637062 |  |  |
| Cannaceae | *Canna indica* L. | GI 557637135 |  |  |
| Strelitziaceae | *Ravenala madagascariensis* Sonn. | GI 563940634 |  |  |
| Marantaceae | *Maranta leuconeura* E.Morren | GI 557637221 |  |  |
