# Supplementary material for: Revisiting the Zingiberales: using multiplexed exon capture to resolve ancient and recent phylogenetic splits in a charismatic plant lineage
Source: PeerJ. 2016 Jan 21;4:e1584. doi: 10.7717/peerj.1584 (PMC4727956; doi:10.7717/peerj.1584)
Supplement: Supplemental Information 3 — Positions within the CDS are appended to the name of the gene. Average length is calculated per individual prior to alignment, or intron removal in the case of plastid genes. [file peerj-04-1584-s003.docx]

| **Gene name and annotation** | **Average Coverage** | **Average Length** | **Reason For Exclusion** | **Final Aligned Length** | **Final Aligned PI sites** |
| --- | --- | --- | --- | --- | --- |
| GSMUA_Achr1T00810_001_40S_ribosomal_protein_S17:0-438 | 51.2369 | 355 | BLAST | NA | NA |
| GSMUA_Achr1T05180_001_Cell_division_protease_ftsH_homolog_5_mitochondrial:738-1215 | 20.9064 | 272 | BLAST | NA | NA |
| GSMUA_Achr1T12760_001_NADH_dehydrogenase_ubiquinone_flavoprotein_1_mitochondrial:375-1476 | 58.757 | 324 | BLAST | NA | NA |
| GSMUA_Achr1T16990_001_40S_ribosomal_protein_S10:331-614 | 29.5233 | 256 | BLAST | NA | NA |
| GSMUA_Achr1T18800_001_Threonyl-tRNA_synthetase_mitochondrial:1095-1317 | 23.7382 | 245 | BLAST | NA | NA |
| GSMUA_Achr1T21900_001_60S_ribosomal_protein_L27a-3:0-243 | 38.0711 | 292 | BLAST | NA | NA |
| GSMUA_Achr2T01220_001_30S_ribosomal_protein_3_chloroplastic:378-657 | 42.6104 | 335 | BLAST | NA | NA |
| GSMUA_Achr2T01640_001_40S_ribosomal_protein_S4:498-936 | 39.1549 | 330 | BLAST | NA | NA |
| GSMUA_Achr2T08960_001_Hypothetical_protein:0-525 | 36.6441 | 258 | BLAST | NA | NA |
| GSMUA_Achr2T16990_001_ADPATP_carrier_protein_1_chloroplastic:639-906 | 35.7079 | 307 | BLAST | NA | NA |
| GSMUA_Achr2T21060_001_Isocitrate_dehydrogenase_NAD_regulatory_subunit_1_mitochondrial:516-756 | 24.6677 | 265 | BLAST | NA | NA |
| GSMUA_Achr2T22080_001_40S_ribosomal_protein_S16:0-210 | 25.2083 | 227 | BLAST | NA | NA |
| GSMUA_Achr3T01440_001_60S_ribosomal_protein_L26-2:0-462 | 45.886 | 288 | BLAST | NA | NA |
| GSMUA_Achr3T20140_001_transposon_protein_putative_CACTA_En_Spm_sub-class_expressed:0-290 | 33.1032 | 260 | BLAST | NA | NA |
| GSMUA_Achr3T26310_001_Glycine_dehydrogenase_decarboxylating_mitochondrial:0-677 | 42.7956 | 295 | BLAST | NA | NA |
| GSMUA_Achr4T00030_001_Glycyl-tRNA_synthetase_1_mitochondrial:960-1366 | 37.2716 | 308 | BLAST | NA | NA |
| GSMUA_Achr4T04240_001_60S_ribosomal_protein_L23:13-298 | 39.2681 | 274 | BLAST | NA | NA |
| GSMUA_Achr4T08480_001_Cell_division_protease_ftsH_homolog_11_chloroplastic_mitochondrial:1830-2112 | 22.7986 | 216 | BLAST | NA | NA |
| GSMUA_Achr4T11890_001_calcium-binding_mitochondrial_protein_anon-60Da_putative_expressed:1436-1608 | 32.1168 | 318 | BLAST | NA | NA |
| GSMUA_Achr4T19830_001_Heat_shock_70_kDa_protein_mitochondrial:798-1419 | 23.7254 | 217 | BLAST | NA | NA |
| GSMUA_Achr4T26620_001_60S_ribosomal_protein_L21-1:0-237 | 30.3798 | 345 | BLAST | NA | NA |
| GSMUA_Achr5T00710_001_Putative_aconitate_hydratase_cytoplasmic:2085-2565 | 62.8748 | 461 | BLAST | NA | NA |
| GSMUA_Achr5T01070_001_60S_ribosomal_protein_L3:501-911 | 48.9493 | 301 | BLAST | NA | NA |
| GSMUA_Achr5T17120_001_Cysteine_synthase_chloroplastic_chromoplastic:387-651 | 43.3195 | 341 | BLAST | NA | NA |
| GSMUA_Achr5T18740_001_60S_ribosomal_protein_L8:440-786 | 70.1945 | 403 | BLAST | NA | NA |
| GSMUA_Achr6T06280_001_60S_ribosomal_protein_L27a-3:123-531 | 40.7298 | 315 | BLAST | NA | NA |
| GSMUA_Achr6T31150_001_60S_ribosomal_protein_L15:247-414 | 29.4814 | 262 | BLAST | NA | NA |
| GSMUA_Achr7T00810_001_Succinate_dehydrogenase_ubiquinone_flavoprotein_subunit_mitochondrial:934-1122 | 23.0254 | 315 | BLAST | NA | NA |
| GSMUA_Achr7T05470_001_Cytochrome_c1-1_heme_protein_mitochondrial:278-558 | 34.273 | 258 | BLAST | NA | NA |
| GSMUA_Achr7T24720_001_Pyruvate_kinase_isozyme_G_chloroplastic:1167-1343 | 41.5436 | 268 | BLAST | NA | NA |
| GSMUA_Achr7T25110_001_60S_ribosomal_protein_L27-3:0-408 | 36.2971 | 270 | BLAST | NA | NA |
| GSMUA_Achr8T01550_001_60S_ribosomal_protein_L10:10-512 | 47.95 | 368 | BLAST | NA | NA |
| GSMUA_Achr8T06120_001_50S_ribosomal_protein_L3-1_chloroplastic:0-379 | 26.2331 | 226 | BLAST | NA | NA |
| GSMUA_Achr8T09450_001_Putative_Solute_carrier_family_35_member_E1:369-828 | 40.8934 | 327 | BLAST | NA | NA |
| GSMUA_Achr8T18300_001_60S_ribosomal_protein_L26-2:0-462 | 45.8681 | 285 | BLAST | NA | NA |
| GSMUA_Achr8T23840_001_60S_ribosomal_protein_L12:0-501 | 57.881 | 412 | BLAST | NA | NA |
| GSMUA_Achr8T30200_001_40S_ribosomal_protein_S9-2:28-410 | 29.1247 | 274 | BLAST | NA | NA |
| GSMUA_Achr9T07000_001_Hypothetical_protein:625-878 | 36.4 | 250 | BLAST | NA | NA |
| GSMUA_Achr9T20320_001_60S_ribosomal_protein_L19-2:235-549 | 49.511 | 364 | BLAST | NA | NA |
| GSMUA_Achr9T21710_001_ATP_synthase_subunit_beta_mitochondrial:1017-1242 | 49.0334 | 580 | BLAST | NA | NA |
| GSMUA_Achr9T21710_001_ATP_synthase_subunit_beta_mitochondrial:1242-1554 | 46.236 | 437 | BLAST | NA | NA |
| GSMUA_Achr9T21710_001_ATP_synthase_subunit_beta_mitochondrial:858-1017 | 28.3174 | 276 | BLAST | NA | NA |
| GSMUA_Achr9T22420_001_Chaperone_protein_ClpB_2:1410-2340 | 31.2425 | 275 | BLAST | NA | NA |
| GSMUA_Achr9T30160_001_Ubiquitin-60S_ribosomal_protein_L40:0-234 | 49.0379 | 296 | BLAST | NA | NA |
| GSMUA_Achr10T10700_001_Protease_Do-like_10_mitochondrial:610-806 | 43.2479 | 298 | BLAST | NA | NA |
| GSMUA_Achr10T12900_001_60S_ribosomal_protein_L10:10-512 | 56.3963 | 410 | BLAST | NA | NA |
| GSMUA_Achr10T14230_001_Putative_mitochondrial_2-oxoglutarate_malate_carrier_protein:269-631 | 44.1061 | 279 | BLAST | NA | NA |
| GSMUA_Achr10T19100_001_ribosomal_protein_L23_family_protein_putative_expressed:0-484 | 40.8498 | 304 | BLAST | NA | NA |
| GSMUA_Achr11T00740_001_60S_ribosomal_protein_L8:152-498 | 61.5246 | 408 | BLAST | NA | NA |
| GSMUA_Achr11T02700_001_40S_ribosomal_protein_S17:0-426 | 56.1486 | 377 | BLAST | NA | NA |
| GSMUA_Achr11T08740_001_Succinate_dehydrogenase_ubiquinone_iron-sulfur_subunit_2_mitochondrial:0-683 | 32.2105 | 282 | BLAST | NA | NA |
| GSMUA_Achr11T09050_001_60S_ribosomal_protein_L3:501-911 | 56.0026 | 318 | BLAST | NA | NA |
| GSMUA_Achr11T10220_001_Putative_mitochondrial_2-oxoglutarate-malate_carrier_protein:266-628 | 37.0433 | 316 | BLAST | NA | NA |
| GSMUA_Achr11T16110_001_Probable_NADH_dehydrogenase_ubiquinone_1_alpha_subcomplex_subunit_5_mitochondrial:0-340 | 47.3632 | 356 | BLAST | NA | NA |
| GSMUA_Achr11T22610_001_60S_ribosomal_protein_L8:0-440 | 35.0194 | 305 | BLAST | NA | NA |
| GSMUA_AchrUn_randomT00030_001_Ubiquitin-40S_ribosomal_protein_S27a-1:0-474 | 66.8384 | 367 | BLAST | NA | NA |
| GSMUA_AchrUn_randomT03040_001_Cell_division_protease_ftsH_homolog_2_chloroplastic:522-836 | 39.6379 | 321 | BLAST | NA | NA |
| GSMUA_AchrUn_randomT08730_001_RuBisCO_large_subunit-binding_protein_subunit_alpha_chloroplastic:994-1446 | 39.6211 | 403 | BLAST | NA | NA |
| GSMUA_AchrUn_randomT10300_001_protein_kinase_APK1B_chloroplast_precursor_putative_expressed:706-1098 | 36.3959 | 269 | BLAST | NA | NA |
| GSMUA_AchrUn_randomT18700_001_Cell_division_protease_ftsH_homolog_8_mitochondrial:1032-1800 | 48.9364 | 344 | BLAST | NA | NA |
| GSMUA_AchrUn_randomT22440_001_ATP-dependent_Clp_protease_ATP-binding_subunit_clpC_homolog_chloroplastic:2937-3177 | 33.2733 | 228 | BLAST | NA | NA |
| GSMUA_AchrUn_randomT26360_001_ATP_synthase_subunit_beta_mitochondrial:1305-1656 | 51.2105 | 454 | BLAST | NA | NA |
| GSMUA_AchrUn_randomT28100_001_Chlorophyll_a-b_binding_protein_CP29.2_chloroplastic:69-441 | 24.1654 | 242 | BLAST | NA | NA |
| GSMUA_Achr6T22780_001_Maturase_K:0-459 | 906.101 | 339 | high coverage | NA | NA |
| GSMUA_Achr6T36680_001_NADPH-quinone_oxidoreductase_subunit_H_chloroplastic:144-762:0 | 976.63 | 265 | high coverage | NA | NA |
| GSMUA_Achr6T36680_001_NADPH-quinone_oxidoreductase_subunit_H_chloroplastic:144-762:3 | 1696.92 | 326 | high coverage | NA | NA |
| GSMUA_Achr10T02890_001_Hypothetical_protein:0-252 | 479.839 | 237 | high coverage | NA | NA |
| GSMUA_Achr11T15530_001_50S_ribosomal_protein_L22_chloroplastic:0-390 | 1410.99 | 381 | high coverage | NA | NA |
| GSMUA_AchrUn_randomT03080_001_Ribosomal_protein_S12_mitochondrial:0-378 | 249.096 | 276 | high coverage | NA | NA |
| GSMUA_AchrUn_randomT05630_001_NADH-ubiquinone_oxidoreductase_27_kDa_subunit:0-573 | 556.039 | 573 | high coverage | NA | NA |
| GSMUA_AchrUn_randomT10060_001_60S_ribosomal_protein_L2_mitochondrial:863-1317 | 306.494 | 460 | high coverage | NA | NA |
| GSMUA_AchrUn_randomT10070_001_Ribosomal_protein_S19_mitochondrial:0-279 | 242.879 | 366 | high coverage | NA | NA |
| GSMUA_AchrUn_randomT10550_001_Apocytochrome_f:517-786 | 675.16 | 242 | high coverage | NA | NA |
| GSMUA_AchrUn_randomT15230_001_ATP_synthase_subunit_alpha_mitochondrial:841-1426 | 232.038 | 273 | high coverage | NA | NA |
| GSMUA_AchrUn_randomT15230_001_ATP_synthase_subunit_alpha_mitochondrial:86-841 | 574.525 | 564 | high coverage | NA | NA |
| GSMUA_AchrUn_randomT21760_001_ATP_synthase_subunit_b_chloroplastic:0-267 | 912.333 | 280 | high coverage | NA | NA |
| GSMUA_AchrUn_randomT21780_001_Hypothetical_protein:0-322 | 199.754 | 181 | high coverage | NA | NA |
| GSMUA_AchrUn_randomT21780_001_Hypothetical_protein:14271-15628 | 306.993 | 203 | high coverage | NA | NA |
| GSMUA_AchrUn_randomT21780_001_Hypothetical_protein:322-550 | 1390.89 | 263 | high coverage | NA | NA |
| GSMUA_AchrUn_randomT21780_001_Hypothetical_protein:550-14271 | 2951.5 | 445 | high coverage | NA | NA |
| GSMUA_AchrUn_randomT21790_001_Photosystem_I_P700_chlorophyll_a_apoprotein_A1:0-196 | 876.13 | 221 | high coverage | NA | NA |
| GSMUA_AchrUn_randomT21790_001_Photosystem_I_P700_chlorophyll_a_apoprotein_A1:196-558 | 2860.15 | 372 | high coverage | NA | NA |
| GSMUA_AchrUn_randomT29040_001_NADPH-quinone_oxidoreductase_subunit_I_chloroplastic:0-369 | 1767.9 | 372 | high coverage | NA | NA |
| GSMUA_Achr5T17250_001_Protein_transport_protein_Sec61_subunit_alpha:0-344:[0-9][0-9]- | 36.5085 | 364 | introduced frame shift | NA | NA |
| GSMUA_Achr5T17250_001_Protein_transport_protein_Sec61_subunit_alpha:0-344:[0-9][0-9][0-9]-344 | 45.9691 | 323 | introduced frame shift | NA | NA |
| GSMUA_Achr5T17250_001_Protein_transport_protein_Sec61_subunit_alpha:1188-1428 | 27.8343 | 254 | introduced frame shift | NA | NA |
| GSMUA_Achr5T17250_001_Protein_transport_protein_Sec61_subunit_alpha:572-927 | 51.1876 | 427 | introduced frame shift | NA | NA |
| GSMUA_Achr1T20200_001_Actin-1:364-978 | 29.6714 | 341 | skewed tree length | NA | NA |
| GSMUA_Achr1T22900_001_Sulfite_reductase_ferredoxin:174-951 | 19.8652 | 264 | skewed tree length | NA | NA |
| GSMUA_Achr1T28070_001_Putative_Importin_subunit_beta-1:0-856 | 28.608 | 244 | skewed tree length | NA | NA |
| GSMUA_Achr1T28070_001_Putative_Importin_subunit_beta-1:856-2324 | 46.5931 | 338 | skewed tree length | NA | NA |
| GSMUA_Achr2T02040_001_Dihydrolipoyl_dehydrogenase_mitochondrial:246-785 | 48.0778 | 329 | skewed tree length | NA | NA |
| GSMUA_Achr2T02040_001_Dihydrolipoyl_dehydrogenase_mitochondrial:785-1407 | 39.0781 | 282 | skewed tree length | NA | NA |
| GSMUA_Achr2T03830_001_Hypothetical_protein:209-411 | 44.4746 | 305 | skewed tree length | NA | NA |
| GSMUA_Achr2T12390_001_Tubulin_alpha-3_chain:1037-1353 | 48.4475 | 334 | skewed tree length | NA | NA |
| GSMUA_Achr2T14040_001_Thiazole_biosynthetic_enzyme_chloroplastic:0-375 | 22.3311 | 250 | skewed tree length | NA | NA |
| GSMUA_Achr2T14200_001_Ribose-phosphate_pyrophosphokinase_3:68-384 | 26.6951 | 316 | skewed tree length | NA | NA |
| GSMUA_Achr3T00530_001_Putative_Ubiquilin-1:241-1015 | 26.8965 | 277 | skewed tree length | NA | NA |
| GSMUA_Achr3T01410_001_DEAD-box_ATP-dependent_RNA_helicase_21:501-999 | 33.1532 | 289 | skewed tree length | NA | NA |
| GSMUA_Achr3T04890_001_LRR_receptor-like_Serine-threonine-protein_kinase_FEI_1:850-1183 | 23.8483 | 264 | skewed tree length | NA | NA |
| GSMUA_Achr3T08780_001_Putative_4-alpha-glucanotransferase:1473-1716 | 30.3487 | 328 | skewed tree length | NA | NA |
| GSMUA_Achr3T18630_001_ATP-dependent_Clp_protease_ATP-binding_subunit_clpA_homolog_CD4B_chloroplastic:1011-1227 | 25.9992 | 385 | skewed tree length | NA | NA |
| GSMUA_Achr4T27850_001_Nuclear_transcription_factor_Y_subunit_B-3:0-386 | 72.3659 | 407 | skewed tree length | NA | NA |
| GSMUA_Achr5T03620_001_6-phosphogluconate_dehydrogenase_decarboxylating:221-1170 | 36.1793 | 323 | skewed tree length | NA | NA |
| GSMUA_Achr5T08520_001_Clathrin_heavy_chain_1:2891-3894 | 57.7728 | 760 | skewed tree length | NA | NA |
| GSMUA_Achr5T08970_001_Putative_Speckle-type_POZ_protein:0-401 | 45.8869 | 360 | skewed tree length | NA | NA |
| GSMUA_Achr5T14620_001_Hypothetical_protein:0-172 | 36.0912 | 288 | skewed tree length | NA | NA |
| GSMUA_Achr5T28260_001_Putative_Zinc_finger_CCCH_domain-containing_protein_66:70-1086 | 53.6057 | 378 | skewed tree length | NA | NA |
| GSMUA_Achr6T12250_001_26S_protease_regulatory_subunit_6B_homolog:150-381 | 47.9889 | 313 | skewed tree length | NA | NA |
| GSMUA_Achr6T36390_001_Chitinase-like_protein_1:0-679 | 35.1145 | 292 | skewed tree length | NA | NA |
| GSMUA_Achr7T08250_001_Putative_Probable_disease_resistance_protein_At4g33300:1083-1439 | 28.0946 | 319 | skewed tree length | NA | NA |
| GSMUA_Achr7T24390_001_Eukaryotic_translation_initiation_factor_1A:0-435 | 57.0108 | 330 | skewed tree length | NA | NA |
| GSMUA_Achr8T00950_001_UDP-glucuronate_4-epimerase_6:606-1332 | 38.0426 | 309 | skewed tree length | NA | NA |
| GSMUA_Achr9T01620_001_Serine_hydroxymethyltransferase_2:0-678 | 56.3328 | 387 | skewed tree length | NA | NA |
| GSMUA_Achr9T03520_001_26S_proteasome_non-ATPase_regulatory_subunit_14:130-308 | 32.4236 | 272 | skewed tree length | NA | NA |
| GSMUA_Achr9T29870_001_ABC_transporter_F_family_member_4:963-1448 | 32.5227 | 282 | skewed tree length | NA | NA |
| GSMUA_Achr10T23290_001_serine-threonine-protein_kinase_HT1_putative_expressed:0-899 | 28.3207 | 248 | skewed tree length | NA | NA |
| GSMUA_Achr10T29540_001_Formate-tetrahydrofolate_ligase:312-524 | 38.0574 | 283 | skewed tree length | NA | NA |
| GSMUA_Achr10T29540_001_Formate-tetrahydrofolate_ligase:524-1359 | 43.6662 | 366 | skewed tree length | NA | NA |
| GSMUA_Achr11T10020_001_Heat_shock_protein_81-3:918-1398 | 43.5256 | 361 | skewed tree length | NA | NA |
| GSMUA_Achr11T17170_001_HEAT_repeat_family_protein_putative_expressed:1777-3543 | 20.1884 | 271 | skewed tree length | NA | NA |
| GSMUA_Achr11T19070_001_Putative_Basic_leucine_zipper_and_W2_domain-containing_protein_2:501-995 | 26.6537 | 277 | skewed tree length | NA | NA |
| GSMUA_Achr11T22900_001_Ubiquitin-fold_modifier-conjugating_enzyme_1:0-395 | 33.1192 | 301 | skewed tree length | NA | NA |
| GSMUA_AchrUn_randomT06180_001_Tubulin_beta-7_chain:696-978 | 83.4275 | 318 | skewed tree length | NA | NA |
| GSMUA_Achr1T00220_001_DEAD-box_ATP-dependent_RNA_helicase_6:804-1056 | 19.553 | 234 |  | 170 | 50 |
| GSMUA_Achr1T02360_001_Probable_aquaporin_PIP2-6:0-292 | 38.8117 | 352 |  | 230 | 77 |
| GSMUA_Achr1T04330_001_Coatomer_subunit_alpha-2:1590-2131 | 25.7 | 289 |  | 158 | 53 |
| GSMUA_Achr1T07110_001_ZOS9-17-C2H2_zinc_finger_protein_expressed:225-674 | 57.4104 | 354 |  | 263 | 42 |
| GSMUA_Achr1T07620_001_Endoplasmin_homolog:1779-2184:0123 | 31.1818 | 278 |  | 380 | 109 |
| GSMUA_Achr1T07620_001_Endoplasmin_homolog:1779-2184:1516 | 42.769 | 289 | merged | NA | NA |
| GSMUA_Achr1T07700_001_Lon_protease_homolog_2_peroxisomal:1523-1737 | 19.619 | 282 |  | 170 | 58 |
| GSMUA_Achr1T08940_001_4-hydroxy-3-methylbut-2-en-1-yl_diphosphate_synthase:783-1014 | 52.1309 | 366 |  | 230 | 66 |
| GSMUA_Achr1T09070_001_Phenylalanine_ammonia-lyase:389-1773 | 28.6982 | 300 |  | 167 | 44 |
| GSMUA_Achr1T10100_001_Putative_Transmembrane_9_superfamily_member_4:771-1077 | 41.9114 | 376 |  | 245 | 71 |
| GSMUA_Achr1T10550_001_26S_proteasome_non-ATPase_regulatory_subunit_1:1470-1691 | 40.2234 | 342 |  | 464 | 142 |
| GSMUA_Achr1T10550_001_26S_proteasome_non-ATPase_regulatory_subunit_1:2183-2700 | 42.6733 | 393 | merged | NA | NA |
| GSMUA_Achr1T12930_001_S-adenosylmethionine_synthase_1:216-1407 | 32.1281 | 329 |  | 194 | 62 |
| GSMUA_Achr1T13970_001_Protease_Do-like_1_chloroplastic:773-972 | 30.2061 | 278 |  | 170 | 43 |
| GSMUA_Achr1T14710_001_T-complex_protein_1_subunit_zeta:936-1141 | 24.5045 | 278 |  | 161 | 43 |
| GSMUA_Achr1T14740_001_Cullin-1:240-426 | 32.6784 | 317 |  | 170 | 44 |
| GSMUA_Achr1T19130_001_Putative_uncharacterized_protein_Sb07g010440:6562-7845:4 | 28.1556 | 380 |  | 497 | 133 |
| GSMUA_Achr1T19130_001_Putative_uncharacterized_protein_Sb07g010440:6562-7845:89 | 24.6845 | 406 | merged | NA | NA |
| GSMUA_Achr1T19130_001_Putative_uncharacterized_protein_Sb07g010440:9120-9525 | 23.8067 | 369 | merged | NA | NA |
| GSMUA_Achr1T22920_001_Probable_cellulose_synthase_A_catalytic_subunit_8_UDP-forming:974-1320 | 45.401 | 336 |  | 218 | 54 |
| GSMUA_Achr1T24870_001_DEAD-box_ATP-dependent_RNA_helicase_24:1215-1518 | 51.4165 | 328 |  | 356 | 107 |
| GSMUA_Achr1T24870_001_DEAD-box_ATP-dependent_RNA_helicase_24:442-1134 | 28.8221 | 250 | merged | NA | NA |
| GSMUA_Achr1T25100_001_heat_shock_protein_binding_protein_putative_expressed:510-728 | 25.8312 | 373 |  | 383 | 105 |
| GSMUA_Achr1T25100_001_heat_shock_protein_binding_protein_putative_expressed:728-906 | 33.9745 | 380 | merged | NA | NA |
| GSMUA_Achr1T25530_001_Probable_pyridoxal_biosynthesis_protein_PDX1.1:0-675 | 30.9489 | 360 |  | 308 | 91 |
| GSMUA_Achr1T27340_001_Uncharacterized_urease_accessory_protein_ureG-like:321-523 | 37.2262 | 330 |  | 203 | 62 |
| GSMUA_Achr2T01410_001_Tubulin_beta-1_chain:559-1035 | 67.8319 | 547 |  | 362 | 114 |
| GSMUA_Achr2T01660_001_ATKINESIN-13A_KINESIN-13A_putative_expressed:1001-1331 | 29.7017 | 253 |  | 188 | 61 |
| GSMUA_Achr2T01800_001_T-complex_protein_1_subunit_beta:635-1121 | 49.857 | 320 |  | 230 | 75 |
| GSMUA_Achr2T01990_001_Protein_translocase_subunit_secA_chloroplastic:657-876 | 33.9486 | 288 |  | 191 | 43 |
| GSMUA_Achr2T03840_001_Pyruvate_kinase_cytosolic_isozyme:489-1035 | 42.3664 | 251 |  | 173 | 50 |
| GSMUA_Achr2T04510_001_Mannosyl-oligosaccharide_12-alpha-mannosidase_MNS1:1320-1491 | 29.0354 | 252 |  | 161 | 49 |
| GSMUA_Achr2T05230_001_Pre-mRNA-processing-splicing_factor_8:2301-2606 | 30.9451 | 322 |  | 1592 | 414 |
| GSMUA_Achr2T05230_001_Pre-mRNA-processing-splicing_factor_8:3852-4106 | 32.9062 | 310 | merged | NA | NA |
| GSMUA_Achr2T05230_001_Pre-mRNA-processing-splicing_factor_8:4106-4723 | 72.3354 | 780 | merged | NA | NA |
| GSMUA_Achr2T05230_001_Pre-mRNA-processing-splicing_factor_8:4830-5085 | 34.739 | 349 | merged | NA | NA |
| GSMUA_Achr2T05230_001_Pre-mRNA-processing-splicing_factor_8:5085-5559 | 41.708 | 339 | merged | NA | NA |
| GSMUA_Achr2T05230_001_Pre-mRNA-processing-splicing_factor_8:5919-6198 | 25.5519 | 271 | merged | NA | NA |
| GSMUA_Achr2T06520_001_Histone_deacetylase_6:312-753 | 40.6362 | 286 |  | 161 | 34 |
| GSMUA_Achr2T06800_001_Ferredoxin-dependent_glutamate_synthase_chloroplastic:2736-2976 | 33.6289 | 385 |  | 212 | 59 |
| GSMUA_Achr2T06890_001_Putative_Magnesium-chelatase_subunit_H:1668-2523 | 61.0222 | 749 |  | 647 | 189 |
| GSMUA_Achr2T08110_001_Calcium-dependent_protein_kinase_SK5:0-556 | 44.6051 | 275 |  | 191 | 51 |
| GSMUA_Achr2T09260_001_E3_ubiquitin-protein_ligase_SINAT5:579-942 | 26.0703 | 306 |  | 173 | 59 |
| GSMUA_Achr2T09300_001_Chlorophyll_a-b_binding_protein_3C_chloroplastic:0-804 | 59.8279 | 746 |  | 671 | 205 |
| GSMUA_Achr2T11540_001_Protein_ETHYLENE_INSENSITIVE_3:0-1815 | 47.1731 | 291 |  | 197 | 58 |
| GSMUA_Achr2T12670_001_37_kDa_inner_envelope_membrane_protein_chloroplastic:263-570 | 46.7461 | 382 |  | 275 | 82 |
| GSMUA_Achr2T13670_001_histone-like_transcription_factor_and_archaeal_histone_putative_expressed:0-417 | 62.9506 | 340 |  | 221 | 54 |
| GSMUA_Achr2T14120_001_Sugar_carrier_protein_C:456-1086 | 38.1269 | 263 |  | 167 | 56 |
| GSMUA_Achr2T14960_001_Chlorophyll_a-b_binding_protein_4_chloroplastic:206-622 | 45.2237 | 316 |  | 224 | 66 |
| GSMUA_Achr2T16160_001_Actin-101:454-1068:0 | 71.5982 | 516 |  | 938 | 262 |
| GSMUA_Achr2T16160_001_Actin-101:454-1068:3 | 57.7281 | 285 | merged | NA | NA |
| GSMUA_Achr2T16160_001_Actin-101:60-454 | 72.767 | 591 | merged | NA | NA |
| GSMUA_Achr2T16710_001_V-type_proton_ATPase_subunit_B2:385-594 | 41.6037 | 348 |  | 191 | 47 |
| GSMUA_Achr2T19080_001_Histone_H2B.6:0-432 | 58.8201 | 386 |  | 272 | 83 |
| GSMUA_Achr2T19320_001_Mitogen-activated_protein_kinase_1:453-786 | 36.1492 | 301 |  | 155 | 39 |
| GSMUA_Achr2T19700_001_Probable_cellulose_synthase_A_catalytic_subunit_1_UDP-forming:2059-2256 | 40.8684 | 341 |  | 404 | 122 |
| GSMUA_Achr2T19700_001_Probable_cellulose_synthase_A_catalytic_subunit_1_UDP-forming:992-1314 | 43.1999 | 352 | merged | NA | NA |
| GSMUA_Achr2T20610_001_Transmembrane_9_superfamily_member_3:1156-1530 | 31.4402 | 297 |  | 416 | 122 |
| GSMUA_Achr2T20610_001_Transmembrane_9_superfamily_member_3:1530-1804 | 35.3814 | 334 | merged | NA | NA |
| GSMUA_Achr3T00140_001_Dolichyl-diphosphooligosaccharide--protein_glycosyltransferase_subunit_STT3:1023-1478 | 33.0443 | 273 |  | 170 | 48 |
| GSMUA_Achr3T01430_001_Vacuolar_protein_sorting-associated_protein_4:512-711 | 33.3775 | 316 |  | 203 | 47 |
| GSMUA_Achr3T01900_001_Sucrose_synthase_2:1008-1182 | 26.9943 | 263 |  | 155 | 47 |
| GSMUA_Achr3T04010_001_Putative_heat_shock_protein_HSP_90-beta-3:1228-1692 | 36.8 | 279 |  | 413 | 129 |
| GSMUA_Achr3T04010_001_Putative_heat_shock_protein_HSP_90-beta-3:549-765 | 32.5051 | 302 | merged | NA | NA |
| GSMUA_Achr3T04370_001_Putative_U5_small_nuclear_ribonucleoprotein_40_kDa_protein:0-514 | 46.6932 | 342 |  | 224 | 64 |
| GSMUA_Achr3T04520_001_Putative_Transmembrane_9_superfamily_member_4:471-1341 | 37.6522 | 282 |  | 179 | 52 |
| GSMUA_Achr3T04920_001_Eukaryotic_translation_initiation_factor_3_subunit_C:1722-2679 | 27.1699 | 276 |  | 167 | 52 |
| GSMUA_Achr3T05060_001_Histone_H4:0-312 | 25.1159 | 306 |  | 218 | 54 |
| GSMUA_Achr3T05800_001_Putative_Ethylene-responsive_transcription_factor_1:280-537 | 22.0681 | 275 |  | 164 | 54 |
| GSMUA_Achr3T05890_001_3-oxoacyl-acyl-carrier-protein_synthase_I_chloroplastic:450-882 | 45.1695 | 410 |  | 266 | 73 |
| GSMUA_Achr3T06150_001_Putative_U4-U6_small_nuclear_ribonucleoprotein_Prp31:228-1121 | 46.5842 | 436 |  | 245 | 77 |
| GSMUA_Achr3T07850_001_Phospholipid_diacylglycerol_acyltransferase_1:693-977 | 43.3477 | 389 |  | 266 | 84 |
| GSMUA_Achr3T10970_001_Heat_shock_protein_81-3:930-1540 | 31.8971 | 315 |  | 155 | 47 |
| GSMUA_Achr3T11550_001_Transketolase_chloroplastic:1299-2256 | 54.7612 | 419 |  | 317 | 111 |
| GSMUA_Achr3T12250_001_lung_seven_transmembrane_domain_containing_protein_putative_expressed:276-1179 | 71.1379 | 614 |  | 515 | 162 |
| GSMUA_Achr3T12630_001_Peptidyl-prolyl_cis-trans_isomerase:0-456 | 23.4992 | 209 |  | 167 | 51 |
| GSMUA_Achr3T12710_001_Probable_pre-mRNA-splicing_factor_ATP-dependent_RNA_helicase:476-1561 | 24.1228 | 305 |  | 185 | 58 |
| GSMUA_Achr3T12780_001_S-adenosylmethionine_synthase:0-1185:2 | 33.8866 | 281 |  | 344 | 111 |
| GSMUA_Achr3T12780_001_S-adenosylmethionine_synthase:0-1185:67 | 29.6497 | 278 | merged | NA | NA |
| GSMUA_Achr3T14360_001_Catalase_isozyme_A:390-1167:1689 | 68.857 | 612 |  | 701 | 235 |
| GSMUA_Achr3T14360_001_Catalase_isozyme_A:390-1167:4 | 49.9715 | 391 | merged | NA | NA |
| GSMUA_Achr3T17720_001_ABC_transporter_B_family_member_20:1393-1622 | 26.8276 | 317 |  | 467 | 148 |
| GSMUA_Achr3T17720_001_ABC_transporter_B_family_member_20:3258-3759 | 64.0986 | 446 | merged | NA | NA |
| GSMUA_Achr3T17740_001_Calcium-transporting_ATPase_1_endoplasmic_reticulum-type:2312-2541 | 26.4015 | 300 |  | 179 | 57 |
| GSMUA_Achr3T19120_001_Probable_Serine-threonine-protein_kinase_At1g01540:1023-1233 | 22.6075 | 307 |  | 173 | 50 |
| GSMUA_Achr3T19370_001_MYB_family_transcription_factor_putative_expressed:0-496 | 55.3253 | 346 |  | 275 | 84 |
| GSMUA_Achr3T19770_001_tyrosine_protein_kinase_domain_containing_protein_putative_expressed:130-450 | 41.7842 | 308 |  | 173 | 47 |
| GSMUA_Achr3T19880_001_Pyrophosphate-energized_vacuolar_membrane_proton_pump:1121-1443 | 41.0938 | 264 |  | 188 | 57 |
| GSMUA_Achr3T23490_001_Tubulin_beta-1_chain:559-1025 | 61.5882 | 529 |  | 347 | 109 |
| GSMUA_Achr3T25040_001_Calmodulin:76-450 | 54.9512 | 347 |  | 230 | 70 |
| GSMUA_Achr3T25050_001_Magnesium-protoporphyrin_IX_monomethyl_ester_oxidative_cyclase_chloroplastic:518-846 | 55.0201 | 387 |  | 287 | 70 |
| GSMUA_Achr3T26080_001_Coatomer_subunit_gamma-2:954-1205 | 32.4126 | 306 |  | 227 | 64 |
| GSMUA_Achr3T27400_001_Probable_voltage-gated_potassium_channel_subunit_beta:132-489 | 27.5082 | 264 |  | 203 | 60 |
| GSMUA_Achr3T27470_001_Caffeic_acid_3-O-methyltransferase:416-727 | 32.2399 | 276 |  | 161 | 47 |
| GSMUA_Achr4T01550_001_Thylakoid_lumenal_19_kDa_protein_chloroplastic:0-729 | 21.3485 | 271 |  | 167 | 48 |
| GSMUA_Achr4T01890_001_Proteasome_subunit_beta_type-3:6-188 | 37.9655 | 342 |  | 173 | 52 |
| GSMUA_Achr4T02120_001_Pentatricopeptide_repeat-containing_protein_At5g10690:731-1225 | 35.2027 | 334 |  | 173 | 52 |
| GSMUA_Achr4T05570_001_Mitogen-activated_protein_kinase_2:432-765 | 37.6519 | 269 |  | 185 | 61 |
| GSMUA_Achr4T05850_001_26S_protease_regulatory_subunit_4_homolog:833-1050 | 41.8395 | 288 |  | 197 | 65 |
| GSMUA_Achr4T06270_001_E3_ubiquitin-protein_ligase_UPL1:8558-9654 | 25.8078 | 259 |  | 161 | 47 |
| GSMUA_Achr4T07140_001_Tryptophan_synthase_beta_chain_2_chloroplastic:225-582 | 37.5462 | 312 |  | 173 | 59 |
| GSMUA_Achr4T08600_001_Eukaryotic_translation_initiation_factor_1A:0-435 | 72.8861 | 422 |  | 332 | 88 |
| GSMUA_Achr4T09920_001_Protein_TOPLESS:2481-2661 | 52.0592 | 307 |  | 176 | 52 |
| GSMUA_Achr4T11560_001_ECT5_putative_expressed:1317-1530 | 29.0674 | 285 |  | 200 | 57 |
| GSMUA_Achr4T11590_001_amino_acid_permease_family_protein_putative_expressed:0-489 | 39.88 | 326 |  | 230 | 63 |
| GSMUA_Achr4T11940_001_Luminal-binding_protein_4:494-709 | 30.2324 | 282 |  | 212 | 64 |
| GSMUA_Achr4T17120_001_Photosystem_I_reaction_center_subunit_II_chloroplastic:0-624 | 17.266 | 210 |  | 164 | 36 |
| GSMUA_Achr4T17710_001_Oryzain_alpha_chain:415-651 | 35.3382 | 328 |  | 191 | 66 |
| GSMUA_Achr4T17930_001_Pre-mRNA_branch_site_p14-like_protein:0-378 | 59.8088 | 369 |  | 278 | 77 |
| GSMUA_Achr4T21470_001_5-methyltetrahydropteroyltriglutamate-homocysteine_methyltransferase:137-491 | 53.1711 | 391 |  | 470 | 147 |
| GSMUA_Achr4T21470_001_5-methyltetrahydropteroyltriglutamate-homocysteine_methyltransferase:1620-1871 | 40.1842 | 283 | merged | NA | NA |
| GSMUA_Achr4T23500_001_RNA_recognition_motif_containing_protein_putative_expressed:37-301 | 36.1481 | 311 |  | 218 | 66 |
| GSMUA_Achr4T24420_001_Putative_Serine-threonine-protein_kinase_HT1:659-834 | 29.8918 | 266 |  | 173 | 52 |
| GSMUA_Achr4T25210_001_Serine-threonine-protein_phosphatase_PP1:178-738 | 33.0362 | 295 |  | 173 | 49 |
| GSMUA_Achr4T25930_001_SNF2_family_N-terminal_domain_containing_protein_expressed:2045-2264 | 27.622 | 317 |  | 161 | 44 |
| GSMUA_Achr4T26250_001_Tubulin_beta-7_chain:664-1341 | 62.62 | 411 |  | 278 | 71 |
| GSMUA_Achr4T28510_001_Pyruvate_kinase_isozyme_A_chloroplastic:476-955 | 38.4354 | 294 |  | 170 | 48 |
| GSMUA_Achr4T29300_001_Serine-threonine-protein_phosphatase_PP1:738-957 | 26.9126 | 321 |  | 176 | 42 |
| GSMUA_Achr4T30080_001_Putative_casein_kinase_II_subunit_beta-4:546-712 | 23.4096 | 288 |  | 164 | 53 |
| GSMUA_Achr4T30650_001_14-3-3-like_protein_GF14-C:0-317 | 46.5053 | 347 |  | 272 | 86 |
| GSMUA_Achr4T31270_001_Glutamate_decarboxylase_1:86-291 | 44.4489 | 279 |  | 197 | 60 |
| GSMUA_Achr4T31670_001_Ferredoxin-NADP_reductase_root_isozyme_chloroplastic:887-1146 | 20.4373 | 237 |  | 155 | 49 |
| GSMUA_Achr4T33040_001_Chlorophyll_a-b_binding_protein_151_chloroplastic:143-795:[0-9]- | 48.5533 | 456 |  | 605 | 194 |
| GSMUA_Achr4T33040_001_Chlorophyll_a-b_binding_protein_151_chloroplastic:143-795:[0-9][0-9][0-9]- | 35.215 | 337 | merged | NA | NA |
| GSMUA_Achr5T00040_001_Hypothetical_protein:164-390 | 28.534 | 343 |  | 182 | 45 |
| GSMUA_Achr5T00120_001_Ethylene_receptor:81-450 | 36.8437 | 291 |  | 161 | 52 |
| GSMUA_Achr5T00290_001_Tubulin_beta-1_chain:559-1035 | 58.8509 | 325 |  | 212 | 60 |
| GSMUA_Achr5T01170_001_Protein_SENSITIVE_TO_PROTON_RHIZOTOXICITY_1:226-1036:1367 | 51.7796 | 335 |  | 419 | 138 |
| GSMUA_Achr5T01170_001_Protein_SENSITIVE_TO_PROTON_RHIZOTOXICITY_1:226-1036:2 | 36.319 | 336 | merged | NA | NA |
| GSMUA_Achr5T02310_001_Putative_Tuftelin-interacting_protein_11:0-1587 | 39.2566 | 330 |  | 164 | 47 |
| GSMUA_Achr5T03040_001_emp24_gp25L_p24_family_protein_putative_expressed:222-409 | 25.9653 | 252 |  | 179 | 52 |
| GSMUA_Achr5T03690_001_Putative_Peptide_transporter_PTR2:1179-1638 | 29.3014 | 320 |  | 353 | 96 |
| GSMUA_Achr5T03690_001_Putative_Peptide_transporter_PTR2:354-896 | 27.5978 | 264 | merged | NA | NA |
| GSMUA_Achr5T04520_001_26S_protease_regulatory_subunit_6A_homolog:288-480 | 30.2824 | 328 |  | 170 | 53 |
| GSMUA_Achr5T06320_001_Probable_histone_deacetylase_19:810-1041 | 24.1175 | 229 |  | 173 | 44 |
| GSMUA_Achr5T09230_001_Chlorophyll_a-b_binding_protein_40_chloroplastic:234-402 | 39.7429 | 222 |  | 164 | 47 |
| GSMUA_Achr5T09710_001_eukaryotic_translation_initiation_factor_5B_putative_expressed:885-1293 | 41.4027 | 305 |  | 194 | 61 |
| GSMUA_Achr5T10720_001_Tubulin_beta-1_chain:725-1088 | 90.8613 | 462 |  | 302 | 64 |
| GSMUA_Achr5T12220_001_DEAD-box_ATP-dependent_RNA_helicase_6:330-565 | 28.784 | 252 |  | 170 | 45 |
| GSMUA_Achr5T13160_001_Pyrophosphate-energized_membrane_proton_pump_3:730-903 | 31.0524 | 321 |  | 164 | 38 |
| GSMUA_Achr5T13570_001_Elongation_factor_G_chloroplastic:247-823:2 | 48.6924 | 432 |  | 563 | 155 |
| GSMUA_Achr5T13570_001_Elongation_factor_G_chloroplastic:247-823:89 | 31.2108 | 281 | merged | NA | NA |
| GSMUA_Achr5T13570_001_Elongation_factor_G_chloroplastic:972-1494 | 26.4606 | 247 | merged | NA | NA |
| GSMUA_Achr5T14780_001_Photosystem_I_reaction_center_subunit_II_chloroplastic:0-624 | 41.2611 | 355 |  | 284 | 72 |
| GSMUA_Achr5T14830_001_Tubulin_alpha-3_chain:528-1037 | 54.4837 | 521 |  | 236 | 65 |
| GSMUA_Achr5T15500_001_Cell_division_cycle_protein_48_homolog:1770-2130 | 36.236 | 340 |  | 719 | 229 |
| GSMUA_Achr5T15500_001_Cell_division_cycle_protein_48_homolog:427-1194:12 | 38.6968 | 479 | merged | NA | NA |
| GSMUA_Achr5T15500_001_Cell_division_cycle_protein_48_homolog:427-1194:4 | 48.1035 | 488 | merged | NA | NA |
| GSMUA_Achr5T16500_001_Putative_Outer_membrane_lipoprotein_blc:0-378 | 15.0463 | 235 |  | 185 | 69 |
| GSMUA_Achr5T18560_001_Phenylalanine_ammonia-lyase_1:380-1770 | 59.651 | 375 |  | 215 | 70 |
| GSMUA_Achr5T18760_001_UDP-glucuronate_4-epimerase_3:0-1380 | 38.0137 | 288 |  | 227 | 77 |
| GSMUA_Achr5T20780_001_Putative_Pre-mRNA-processing_factor_6:922-2191:2 | 31.3016 | 389 |  | 362 | 98 |
| GSMUA_Achr5T20780_001_Putative_Pre-mRNA-processing_factor_6:922-2191:5 | 37.9973 | 375 | merged | NA | NA |
| GSMUA_Achr5T21370_001_Putative_Structural_maintenance_of_chromosomes_protein_1A:1415-1686 | 40.7027 | 335 |  | 209 | 58 |
| GSMUA_Achr5T23480_001_Pyrophosphate-energized_vacuolar_membrane_proton_pump:238-813 | 27.8043 | 310 |  | 155 | 46 |
| GSMUA_Achr5T23640_001_Diaminopimelate_decarboxylase_1_chloroplastic:1020-1206 | 20.7315 | 285 |  | 158 | 50 |
| GSMUA_Achr5T25020_001_Serine-threonine-protein_kinase_PBS1:517-909 | 49.0722 | 362 |  | 227 | 74 |
| GSMUA_Achr5T25450_001_Serine-threonine_protein_phosphatase_2A_59_kDa_regulatory_subunit_B_gamma_isoform:264-1026:1 | 28.0892 | 360 |  | 473 | 148 |
| GSMUA_Achr5T25450_001_Serine-threonine_protein_phosphatase_2A_59_kDa_regulatory_subunit_B_gamma_isoform:264-1026:45 | 38.4291 | 420 | merged | NA | NA |
| GSMUA_Achr5T26680_001_Tubulin_beta-1_chain:0-177 | 32.2094 | 320 |  | 461 | 138 |
| GSMUA_Achr5T26680_001_Tubulin_beta-1_chain:559-1035 | 66.1313 | 420 | merged | NA | NA |
| GSMUA_Achr5T26800_001_Copper-transporting_ATPase_RAN1:1610-1953 | 40.1488 | 316 |  | 194 | 57 |
| GSMUA_Achr5T26990_001_Elongation_factor_TS:2399-2733 | 24.1412 | 255 |  | 167 | 61 |
| GSMUA_Achr5T28610_001_ADP-ribosylation_factor_GTPase-activating_protein_AGD7:54-527 | 31.7676 | 228 |  | 161 | 43 |
| GSMUA_Achr6T01660_001_Transcriptional_corepressor_LEUNIG:1440-1683 | 39.8729 | 289 |  | 170 | 51 |
| GSMUA_Achr6T01750_001_Eukaryotic_initiation_factor_4A-1:606-1242 | 33.898 | 309 |  | 206 | 66 |
| GSMUA_Achr6T02020_001_Elongation_factor_1-alpha_putative_expressed:199-675 | 66.3373 | 531 |  | 320 | 94 |
| GSMUA_Achr6T03030_001_expressed_protein:780-1713 | 35.6029 | 326 |  | 188 | 50 |
| GSMUA_Achr6T04090_001_Elongation_factor_1-alpha:21-552 | 32.068 | 347 |  | 161 | 52 |
| GSMUA_Achr6T06520_001_Serine_hydroxymethyltransferase_1:0-389 | 41.869 | 336 |  | 353 | 117 |
| GSMUA_Achr6T06520_001_Serine_hydroxymethyltransferase_1:389-600 | 45.2208 | 312 | merged | NA | NA |
| GSMUA_Achr6T06690_001_Elongation_factor_Tu_chloroplastic:312-627 | 36.7238 | 262 |  | 173 | 46 |
| GSMUA_Achr6T06830_001_Protein_transport_protein_Sec61_subunit_alpha:146-374 | 32.9447 | 349 |  | 167 | 49 |
| GSMUA_Achr6T07940_001_Glutamate-1-semialdehyde_21-aminomutase_chloroplastic:357-1425 | 30.6084 | 297 |  | 182 | 58 |
| GSMUA_Achr6T08040_001_ATP-dependent_Clp_protease_ATP-binding_subunit_clpA_homolog_CD4A_chloroplastic:453-671 | 31.8738 | 411 |  | 416 | 139 |
| GSMUA_Achr6T08040_001_ATP-dependent_Clp_protease_ATP-binding_subunit_clpA_homolog_CD4A_chloroplastic:837-1530 | 51.2816 | 311 | merged | NA | NA |
| GSMUA_Achr6T09310_001_calpain_putative_expressed:6186-6492 | 42.6014 | 344 |  | 257 | 84 |
| GSMUA_Achr6T10120_001_paramyosin_putative_expressed:855-2473 | 24.8411 | 282 |  | 164 | 54 |
| GSMUA_Achr6T10570_001_Putative_MYST-like_histone_acetyltransferase_1:258-471 | 33.8669 | 335 |  | 191 | 49 |
| GSMUA_Achr6T10590_001_ABC_transporter_F_family_member_5:786-1128 | 30.2174 | 311 |  | 173 | 55 |
| GSMUA_Achr6T11210_001_Deoxyhypusine_synthase:805-1041 | 25.0874 | 274 |  | 152 | 49 |
| GSMUA_Achr6T11910_001_Putative_Protein_ALWAYS_EARLY_3:1908-2087 | 33.3474 | 277 |  | 158 | 52 |
| GSMUA_Achr6T15480_001_Serine-threonine-protein_kinase_PBS1:478-870 | 41.3928 | 306 |  | 179 | 50 |
| GSMUA_Achr6T16370_001_Chalcone_synthase_2:178-669 | 29.1014 | 238 |  | 167 | 50 |
| GSMUA_Achr6T16670_001_Chaperone_protein_ClpB_2:1626-2553 | 55.7384 | 385 |  | 278 | 84 |
| GSMUA_Achr6T17750_001_Serine_hydroxymethyltransferase_1:987-1393 | 17.5804 | 262 |  | 179 | 53 |
| GSMUA_Achr6T18780_001_Patellin-3:531-1215 | 45.5412 | 322 |  | 215 | 70 |
| GSMUA_Achr6T18890_001_V-type_proton_ATPase_16_kDa_proteolipid_subunit_c1_c3_c5:76-362 | 52.7428 | 340 |  | 227 | 65 |
| GSMUA_Achr6T19490_001_Carbamoyl-phosphate_synthase_small_chain:750-1098 | 54.9136 | 393 |  | 233 | 69 |
| GSMUA_Achr6T19990_001_Tubulin_beta-1_chain:394-664 | 37.5556 | 271 |  | 374 | 105 |
| GSMUA_Achr6T19990_001_Tubulin_beta-1_chain:855-1071 | 44.2328 | 315 | merged | NA | NA |
| GSMUA_Achr6T20210_001_expressed_protein:3151-3751 | 43.5445 | 346 |  | 230 | 62 |
| GSMUA_Achr6T23120_001_Probable_E3_ubiquitin-protein_ligase_ARI2:1046-1595 | 34.8159 | 276 |  | 497 | 145 |
| GSMUA_Achr6T23120_001_Probable_E3_ubiquitin-protein_ligase_ARI2:565-1046 | 51.6603 | 414 | merged | NA | NA |
| GSMUA_Achr6T23290_001_T-complex_protein_1_subunit_gamma:513-791 | 29.1422 | 291 |  | 197 | 55 |
| GSMUA_Achr6T28370_001_Stromal_70_kDa_heat_shock-related_protein_chloroplastic:1058-1248 | 22.1047 | 238 |  | 407 | 128 |
| GSMUA_Achr6T28370_001_Stromal_70_kDa_heat_shock-related_protein_chloroplastic:1248-1647 | 46.7396 | 322 | merged | NA | NA |
| GSMUA_Achr6T31540_001_Hypothetical_protein:442-790 | 37.946 | 324 |  | 245 | 78 |
| GSMUA_Achr6T31810_001_Cellulose_synthase_A_catalytic_subunit_4_UDP-forming:806-1152 | 21.8163 | 234 |  | 170 | 35 |
| GSMUA_Achr6T31900_001_Calcium-dependent_protein_kinase_3:36-412 | 51.6666 | 322 |  | 245 | 63 |
| GSMUA_Achr6T32910_001_Putative_Zinc_finger_CCCH_domain-containing_protein_66:0-1076 | 55.9515 | 347 |  | 266 | 91 |
| GSMUA_Achr6T33650_001_Cell_division_protease_ftsH_homolog_7_chloroplastic:1083-1407 | 31.9559 | 293 |  | 176 | 62 |
| GSMUA_Achr6T33660_001_Phosphomethylpyrimidine_synthase:1404-2081 | 84.3637 | 705 |  | 803 | 247 |
| GSMUA_Achr6T33660_001_Phosphomethylpyrimidine_synthase:577-1404 | 51.8141 | 355 | merged | NA | NA |
| GSMUA_Achr6T33760_001_Pleckstrin_homology_domain-containing_protein_1:0-471 | 38.1825 | 285 |  | 188 | 49 |
| GSMUA_Achr6T33840_001_Alpha-glucan_phosphorylase_H_isozyme:372-549 | 27.7129 | 291 |  | 176 | 49 |
| GSMUA_Achr6T33940_001_26S_proteasome_non-ATPase_regulatory_subunit_14:399-576 | 39.2312 | 377 |  | 173 | 49 |
| GSMUA_Achr6T33980_001_Mannose-1-phosphate_guanyltransferase_beta:414-762 | 16.3061 | 296 |  | 167 | 57 |
| GSMUA_Achr6T36560_001_Probable_methyltransferase_PMT8:612-1207 | 62.8666 | 476 |  | 368 | 111 |
| GSMUA_Achr7T02100_001_Probable_cellulose_synthase_A_catalytic_subunit_5_UDP-forming:2691-3276 | 53.2966 | 398 |  | 221 | 66 |
| GSMUA_Achr7T05110_001_Probable_GDP-L-fucose_synthase_1:0-993 | 29.9595 | 270 |  | 197 | 58 |
| GSMUA_Achr7T05550_001_Probable_receptor-like_protein_kinase_At2g42960:351-707 | 39.2916 | 356 |  | 224 | 69 |
| GSMUA_Achr7T10170_001_Phospholipase_D_alpha_1:801-1189 | 22.6062 | 216 |  | 161 | 52 |
| GSMUA_Achr7T10410_001_Actin-101:454-1068 | 56.3976 | 341 |  | 641 | 188 |
| GSMUA_Achr7T10410_001_Actin-101:60-454 | 72.3478 | 606 | merged | NA | NA |
| GSMUA_Achr7T14490_001_ABC_transporter_E_family_member_2:996-1182 | 23.8036 | 289 |  | 173 | 54 |
| GSMUA_Achr7T15160_001_Heat_shock_cognate_70_kDa_protein:396-671 | 31.2545 | 239 |  | 383 | 122 |
| GSMUA_Achr7T15160_001_Heat_shock_cognate_70_kDa_protein:747-1074 | 46.8853 | 358 | merged | NA | NA |
| GSMUA_Achr7T15260_001_Histone_H2B.6:0-459 | 39.0125 | 252 |  | 179 | 58 |
| GSMUA_Achr7T15350_001_Ankyrin_repeat-containing_protein_At3g12360:0-439 | 21.7158 | 245 |  | 338 | 114 |
| GSMUA_Achr7T15350_001_Ankyrin_repeat-containing_protein_At3g12360:439-849 | 41.7877 | 283 | merged | NA | NA |
| GSMUA_Achr7T15530_001_PHD_finger_protein_At5g26210:477-729 | 41.1275 | 340 |  | 224 | 75 |
| GSMUA_Achr7T17620_001_26S_protease_regulatory_subunit_S10B_homolog_B:834-1059 | 22.3998 | 271 |  | 164 | 46 |
| GSMUA_Achr7T17740_001_Putative_U-box_domain-containing_protein_13:3352-5566 | 36.4717 | 438 |  | 161 | 50 |
| GSMUA_Achr7T18320_001_Trans-cinnamate_4-monooxygenase:850-1446 | 30.5434 | 256 |  | 188 | 58 |
| GSMUA_Achr7T20920_001_Probable_WRKY_transcription_factor_19:162-586 | 34.8385 | 325 |  | 164 | 55 |
| GSMUA_Achr7T21280_001_Proteasome_subunit_alpha_type-4:0-564 | 28.4967 | 274 |  | 158 | 47 |
| GSMUA_Achr7T21790_001_Probable_methylenetetrahydrofolate_reductase:0-222 | 28.8378 | 252 |  | 158 | 43 |
| GSMUA_Achr7T21890_001_23-bisphosphoglycerate-independent_phosphoglycerate_mutase:645-1070 | 48.3826 | 344 |  | 227 | 76 |
| GSMUA_Achr7T21960_001_26S_protease_regulatory_subunit_6B_homolog:22-693 | 59.5629 | 371 |  | 500 | 145 |
| GSMUA_Achr7T21960_001_26S_protease_regulatory_subunit_6B_homolog:693-924 | 46.2754 | 349 | merged | NA | NA |
| GSMUA_Achr7T22040_001_Vacuolar-sorting_receptor_1:301-994 | 54.9125 | 439 |  | 320 | 106 |
| GSMUA_Achr7T22310_001_PRA1_family_protein_B4:132-567 | 37.8631 | 300 |  | 215 | 88 |
| GSMUA_Achr7T23140_001_Probable_ion_channel_POLLUX:2289-2553 | 32.8482 | 268 |  | 161 | 41 |
| GSMUA_Achr8T00820_001_Acyl-acyl-carrier-protein_desaturase_chloroplastic:48-334 | 39.5932 | 293 |  | 194 | 60 |
| GSMUA_Achr8T02520_001_Mannose-1-phosphate_guanyltransferase_beta:723-1392 | 37.1902 | 290 |  | 194 | 61 |
| GSMUA_Achr8T04480_001_Probable_histone_H2A_variant_3:108-405 | 54.2054 | 343 |  | 242 | 84 |
| GSMUA_Achr8T06200_001_S-adenosylmethionine_synthase:0-1182 | 48.6756 | 397 |  | 212 | 67 |
| GSMUA_Achr8T07200_001_DNA_damage-binding_protein_1:1776-2051 | 32.3106 | 296 |  | 197 | 60 |
| GSMUA_Achr8T07340_001_Polyubiquitin:0-303 | 40.4666 | 268 |  | 182 | 64 |
| GSMUA_Achr8T08130_001_Uncharacterized_protein_At5g49945:0-1179 | 39.902 | 327 |  | 209 | 74 |
| GSMUA_Achr8T08750_001_Acyl-acyl-carrier-protein_desaturase_chloroplastic:125-624 | 52.8212 | 381 |  | 275 | 87 |
| GSMUA_Achr8T08980_001_Serine-threonine_protein_phosphatase_2A_57_kDa_regulatory_subunit_B_theta_isoform:90-459 | 25.9443 | 352 |  | 170 | 41 |
| GSMUA_Achr8T09960_001_Probable_chromatin-remodeling_complex_ATPase_chain:1584-1805 | 33.7529 | 336 |  | 191 | 60 |
| GSMUA_Achr8T10620_001_Probable_ATP-citrate_synthase:1640-1925 | 34.7081 | 264 |  | 167 | 50 |
| GSMUA_Achr8T11090_001_glycosyl_transferase_8_domain_containing_protein_putative_expressed:143-402 | 31.5189 | 308 |  | 173 | 57 |
| GSMUA_Achr8T12670_001_Putative_Pre-mRNA-processing_factor_39:863-1224 | 58.0385 | 390 |  | 245 | 47 |
| GSMUA_Achr8T13360_001_Catalase_isozyme_2:390-1167 | 35.1569 | 357 |  | 239 | 82 |
| GSMUA_Achr8T15940_001_kinesin_motor_domain_containing_protein_putative_expressed:1268-1487 | 28.9424 | 363 |  | 194 | 56 |
| GSMUA_Achr8T16150_001_Actin-7:60-454 | 43.7456 | 406 |  | 176 | 53 |
| GSMUA_Achr8T16590_001_T-complex_protein_1_subunit_eta:147-408 | 38.4413 | 304 |  | 203 | 57 |
| GSMUA_Achr8T17490_001_Adenosylhomocysteinase:426-963 | 43.5342 | 297 |  | 206 | 68 |
| GSMUA_Achr8T19830_001_hAT_dimerisation_domain-containing_protein_putative_expressed:67-1684 | 25.956 | 273 |  | 161 | 40 |
| GSMUA_Achr8T20830_001_Heat_shock_cognate_70_kDa_protein:214-1947:1 | 57.7661 | 348 |  | 1412 | 480 |
| GSMUA_Achr8T20830_001_Heat_shock_cognate_70_kDa_protein:214-1947:5 | 65.0006 | 864 | merged | NA | NA |
| GSMUA_Achr8T20830_001_Heat_shock_cognate_70_kDa_protein:214-1947:78 | 59.4001 | 621 | merged | NA | NA |
| GSMUA_Achr8T21320_001_CBL-interacting_protein_kinase_18:224-676 | 32.8981 | 267 |  | 191 | 67 |
| GSMUA_Achr8T23480_001_Probable_LRR_receptor-like_Serine-threonine-protein_kinase_At5g10290:1108-1503 | 70.015 | 461 |  | 563 | 172 |
| GSMUA_Achr8T23480_001_Probable_LRR_receptor-like_Serine-threonine-protein_kinase_At5g10290:766-1108 | 43.3362 | 349 | merged | NA | NA |
| GSMUA_Achr8T24130_001_Geranylgeranyl_diphosphate_reductase_chloroplastic:0-648 | 22.3699 | 225 |  | 551 | 156 |
| GSMUA_Achr8T24130_001_Geranylgeranyl_diphosphate_reductase_chloroplastic:648-932 | 32.5243 | 302 | merged | NA | NA |
| GSMUA_Achr8T24130_001_Geranylgeranyl_diphosphate_reductase_chloroplastic:932-1368 | 51.8694 | 338 | merged | NA | NA |
| GSMUA_Achr8T24670_001_Eukaryotic_initiation_factor_4A-15:175-606 | 68.2076 | 467 |  | 512 | 155 |
| GSMUA_Achr8T24670_001_Eukaryotic_initiation_factor_4A-15:714-1131 | 35.3035 | 282 | merged | NA | NA |
| GSMUA_Achr8T24860_001_expressed_protein:3277-5491:1 | 57.2892 | 503 |  | 707 | 235 |
| GSMUA_Achr8T24860_001_expressed_protein:3277-5491:2 | 31.8607 | 345 | merged | NA | NA |
| GSMUA_Achr8T24860_001_expressed_protein:5491-5946 | 36.7567 | 304 | merged | NA | NA |
| GSMUA_Achr8T29000_001_frigida_putative_expressed:0-1039 | 42.2521 | 356 |  | 197 | 69 |
| GSMUA_Achr8T29950_001_Probable_glutamyl_endopeptidase_chloroplastic:1553-2012 | 23.9353 | 318 |  | 170 | 54 |
| GSMUA_Achr8T31320_001_RNA_recognition_motif_containing_protein_putative_expressed:198-682 | 55.6361 | 394 |  | 239 | 71 |
| GSMUA_Achr8T31890_001_ATP-dependent_RNA_helicase-like_protein_DB10:717-1068 | 53.9632 | 412 |  | 308 | 102 |
| GSMUA_Achr8T32300_001_PHD_finger_protein_At5g26210:133-362 | 50.5188 | 267 |  | 182 | 47 |
| GSMUA_Achr8T33760_001_AAA-type_ATPase_family_protein_putative_expressed:2712-2897 | 42.0151 | 338 |  | 182 | 49 |
| GSMUA_Achr9T00100_001_Mitogen-activated_protein_kinase_kinase_1:180-393 | 28.3954 | 362 |  | 206 | 54 |
| GSMUA_Achr9T01630_001_Adenosylhomocysteinase:531-1068 | 43.1218 | 277 |  | 182 | 55 |
| GSMUA_Achr9T03250_001_Vacuolar_protein_sorting-associated_protein_2_homolog_1:273-534 | 50.139 | 326 |  | 230 | 71 |
| GSMUA_Achr9T03960_001_Heat_shock_cognate_70_kDa_protein:0-214 | 36.7115 | 298 |  | 200 | 64 |
| GSMUA_Achr9T04060_001_Probable_histone_H2B.1:66-354 | 53.2875 | 361 |  | 284 | 80 |
| GSMUA_Achr9T05610_001_Ras-related_protein_Rab11D:0-221 | 44.886 | 283 |  | 407 | 121 |
| GSMUA_Achr9T05610_001_Ras-related_protein_Rab11D:221-648 | 40.5831 | 304 | merged | NA | NA |
| GSMUA_Achr9T05910_001_protein_kinase_putative_expressed:449-1453 | 43.983 | 333 |  | 200 | 53 |
| GSMUA_Achr9T06900_001_Hypothetical_protein:16-414 | 39.4483 | 321 |  | 257 | 76 |
| GSMUA_Achr9T07750_001_Stromal_70_kDa_heat_shock-related_protein_chloroplastic:0-369 | 19.1783 | 223 |  | 149 | 47 |
| GSMUA_Achr9T11540_001_Probable_potassium_transporter_14:1174-1429 | 37.0186 | 316 |  | 200 | 59 |
| GSMUA_Achr9T12260_001_Probable_cellulose_synthase_A_catalytic_subunit_3_UDP-forming:2835-3255 | 40.2706 | 318 |  | 182 | 56 |
| GSMUA_Achr9T12680_001_Putative_heat_shock_protein_HSP_90-beta-3:1228-1677 | 36.365 | 324 |  | 230 | 74 |
| GSMUA_Achr9T15340_001_Hypothetical_protein:0-1425 | 68.3005 | 432 |  | 326 | 94 |
| GSMUA_Achr9T17280_001_Calmodulin-3:76-450 | 46.4847 | 215 |  | 176 | 56 |
| GSMUA_Achr9T17850_001_Putative_zinc_finger_C3HC4_type_domain_containing_protein_expressed:5151-5379 | 26.3873 | 284 |  | 167 | 53 |
| GSMUA_Achr9T19880_001_expressed_protein:6-818 | 30.6853 | 263 |  | 182 | 62 |
| GSMUA_Achr9T20870_001_Histone_H3.3:138-342 | 29.048 | 269 |  | 182 | 58 |
| GSMUA_Achr9T23240_001_RuBisCO_large_subunit-binding_protein_subunit_beta_chloroplastic:612-855 | 39.5344 | 372 |  | 197 | 60 |
| GSMUA_Achr9T23340_001_Cell_division_cycle_5-like_protein:177-1899 | 74.9838 | 431 |  | 308 | 95 |
| GSMUA_Achr9T23710_001_Luminal-binding_protein_5:712-1192 | 47.3559 | 373 |  | 290 | 96 |
| GSMUA_Achr9T23810_001_DEAD-box_ATP-dependent_RNA_helicase_34:0-257 | 52.0465 | 367 |  | 230 | 70 |
| GSMUA_Achr9T26220_001_Hypothetical_protein:222-558 | 41.4336 | 334 |  | 203 | 54 |
| GSMUA_Achr9T27600_001_Ribose-phosphate_pyrophosphokinase_4:116-432 | 26.8408 | 297 |  | 167 | 47 |
| GSMUA_Achr9T29080_001_Putative_phagocytic_receptor_1b:1077-1562 | 19.3788 | 205 |  | 155 | 51 |
| GSMUA_Achr9T29100_001_Peptide_transporter_PTR2:381-938 | 29.2295 | 338 |  | 197 | 56 |
| GSMUA_Achr10T00180_001_phosphoesterase_family_protein_putative_expressed:0-496 | 18.5316 | 217 |  | 164 | 58 |
| GSMUA_Achr10T01510_001_Calmodulin:76-450 | 56.0572 | 418 |  | 356 | 117 |
| GSMUA_Achr10T01790_001_Developmentally-regulated_GTP-binding_protein_2:215-396 | 23.6724 | 316 |  | 179 | 45 |
| GSMUA_Achr10T03250_001_Elongation_factor_1-alpha:417-654 | 36.0618 | 276 |  | 209 | 70 |
| GSMUA_Achr10T03520_001_Heat_shock_protein_81-2:705-1542 | 54.5462 | 526 |  | 344 | 119 |
| GSMUA_Achr10T03730_001_Actin-2:60-454 | 56.8018 | 587 |  | 266 | 76 |
| GSMUA_Achr10T05860_001_TBC_domain_containing_protein_expressed:1267-1692 | 43.8025 | 359 |  | 248 | 85 |
| GSMUA_Achr10T06510_001_WD_domain_G-beta_repeat_domain_containing_protein_expressed:2678-4068 | 48.9056 | 324 |  | 236 | 71 |
| GSMUA_Achr10T07400_001_SKP1-like_protein_1A:0-306 | 38.9388 | 290 |  | 203 | 60 |
| GSMUA_Achr10T07890_001_ankyrin_repeat_domain_containing_protein_expressed:0-1012 | 35.3323 | 290 |  | 194 | 68 |
| GSMUA_Achr10T09380_001_Digalactosyldiacylglycerol_synthase_1_chloroplastic:1254-1497 | 27.3203 | 290 |  | 215 | 61 |
| GSMUA_Achr10T12360_001_Glyceraldehyde-3-phosphate_dehydrogenase_A_chloroplastic:156-461 | 33.2634 | 304 |  | 167 | 46 |
| GSMUA_Achr10T12740_001_UDP-sulfoquinovose_synthase_chloroplastic:0-771 | 23.2092 | 266 |  | 158 | 50 |
| GSMUA_Achr10T13400_001_Calcium-transporting_ATPase_1_endoplasmic_reticulum-type:684-951 | 39.3811 | 265 |  | 179 | 55 |
| GSMUA_Achr10T16310_001_expressed_protein:1844-2453 | 48.2358 | 413 |  | 473 | 128 |
| GSMUA_Achr10T16310_001_expressed_protein:843-1113 | 41.2652 | 309 | merged | NA | NA |
| GSMUA_Achr10T17010_001_Putative_E3_ubiquitin-protein_ligase_UPL1:8294-9390:7 | 29.1751 | 343 |  | 665 | 206 |
| GSMUA_Achr10T17010_001_Putative_E3_ubiquitin-protein_ligase_UPL1:8294-9390:9 | 21.9448 | 261 | merged | NA | NA |
| GSMUA_Achr10T17330_001_Calnexin_homolog_1:151-1278:1 | 42.3311 | 297 |  | 1145 | 404 |
| GSMUA_Achr10T17330_001_Calnexin_homolog_1:151-1278:4 | 78.5617 | 468 | merged | NA | NA |
| GSMUA_Achr10T17710_001_EF_hand_family_protein_expressed:0-474 | 24.8652 | 332 |  | 275 | 92 |
| GSMUA_Achr10T18810_001_Probable_methyltransferase_PMT2:0-645 | 31.5571 | 265 |  | 164 | 44 |
| GSMUA_Achr10T19930_001_Ribulose_bisphosphate_carboxylase_small_chain_chloroplastic:246-477 | 36.2272 | 289 |  | 224 | 66 |
| GSMUA_Achr10T20580_001_Proteasome_subunit_alpha_type-1-A:351-798 | 20.3119 | 219 |  | 173 | 51 |
| GSMUA_Achr10T21400_001_Elongation_factor_2:1950-2316 | 34.8554 | 338 |  | 563 | 158 |
| GSMUA_Achr10T21400_001_Elongation_factor_2:702-1950 | 36.8178 | 510 | merged | NA | NA |
| GSMUA_Achr10T21400_001_Elongation_factor_2:91-702 | 46.7248 | 330 | merged | NA | NA |
| GSMUA_Achr10T22980_001_Elongation_factor_1-alpha:103-570 | 67.5662 | 509 |  | 377 | 108 |
| GSMUA_Achr10T23400_001_Coatomer_subunit_beta-1:1884-2394 | 31.4225 | 281 |  | 197 | 64 |
| GSMUA_Achr10T24850_001_Riboflavin_biosynthesis_protein_ribBA_chloroplastic:1110-1407 | 47.2325 | 367 |  | 293 | 105 |
| GSMUA_Achr10T26630_001_IWS1_C-terminus_family_protein_putative_expressed:624-845 | 29.9572 | 301 |  | 197 | 55 |
| GSMUA_Achr10T27140_001_26S_protease_regulatory_subunit_8_homolog_A:755-912 | 31.7297 | 273 |  | 155 | 45 |
| GSMUA_Achr10T27330_001_Protein_GIGANTEA:1293-2829 | 42.4491 | 337 |  | 206 | 70 |
| GSMUA_Achr10T28040_001_Putative_Transmembrane_9_superfamily_member_2:1333-2046:1 | 44.4299 | 373 |  | 392 | 122 |
| GSMUA_Achr10T28040_001_Putative_Transmembrane_9_superfamily_member_2:1333-2046:3 | 28.8174 | 235 | merged | NA | NA |
| GSMUA_Achr10T28260_001_Lysyl-tRNA_synthetase:897-1180 | 28.3403 | 301 |  | 158 | 50 |
| GSMUA_Achr10T29400_001_Ubiquitin-activating_enzyme_E1_2:1308-2177 | 49.3064 | 415 |  | 341 | 119 |
| GSMUA_Achr10T30470_001_Chlorophyll_a-b_binding_protein_CP24_10A_chloroplastic:0-450 | 11.866 | 296 |  | 176 | 47 |
| GSMUA_Achr10T31340_001_Probable_phosphatidylinositol_4-kinase_type_2-beta_At1g26270:439-996 | 63.0907 | 420 |  | 311 | 93 |
| GSMUA_Achr10T31490_001_Chloride_channel_protein_CLC-f:369-772 | 46.8063 | 417 |  | 443 | 125 |
| GSMUA_Achr10T31490_001_Chloride_channel_protein_CLC-f:772-967 | 24.3662 | 318 | merged | NA | NA |
| GSMUA_Achr11T00490_001_Ubiquitin-fold_modifier-conjugating_enzyme_1:0-395 | 39.2091 | 385 |  | 176 | 50 |
| GSMUA_Achr11T01900_001_Protochlorophyllide_reductase_B_chloroplastic:676-1035 | 32.6306 | 274 |  | 185 | 48 |
| GSMUA_Achr11T03500_001_Putative_Methionine_gamma-lyase:673-1267 | 32.0773 | 288 |  | 194 | 55 |
| GSMUA_Achr11T04800_001_Pto-interacting_protein_1:463-737 | 47.0674 | 364 |  | 257 | 83 |
| GSMUA_Achr11T05030_001_Probable_ATP-citrate_synthase:1466-1751 | 31.4744 | 298 |  | 185 | 57 |
| GSMUA_Achr11T08060_001_V-type_proton_ATPase_catalytic_subunit_A:1098-1293 | 48.0692 | 333 |  | 191 | 52 |
| GSMUA_Achr11T10550_001_Probable_methylenetetrahydrofolate_reductase:426-600 | 26.0917 | 301 |  | 161 | 46 |
| GSMUA_Achr11T15050_001_Proteasome_subunit_beta_type-4:459-645 | 36.6606 | 357 |  | 185 | 61 |
| GSMUA_Achr11T17980_001_Putative_Protein_transport_protein_SEC23:438-2259 | 23.426 | 259 |  | 149 | 46 |
| GSMUA_Achr11T19060_001_Putative_CUB_and_sushi_domain-containing_protein_2:0-435 | 33.1012 | 232 |  | 170 | 58 |
| GSMUA_Achr11T19850_001_Vacuolar-sorting_receptor_1:298-991 | 71.1959 | 509 |  | 344 | 110 |
| GSMUA_Achr11T24320_001_Ribulose_bisphosphate_carboxylase_oxygenase_activase_2_chloroplastic:557-1030 | 32.4018 | 299 |  | 167 | 39 |
| GSMUA_Achr11T25560_001_Serine-threonine-protein_phosphatase_BSL1_homolog:234-434 | 23.9934 | 293 |  | 173 | 46 |
| GSMUA_AchrUn_randomT01480_001_Clathrin_heavy_chain_1:2876-3879:1 | 44.1161 | 349 |  | 650 | 186 |
| GSMUA_AchrUn_randomT01480_001_Clathrin_heavy_chain_1:2876-3879:3 | 63.0585 | 347 | merged | NA | NA |
| GSMUA_AchrUn_randomT01480_001_Clathrin_heavy_chain_1:3879-4365 | 37.9983 | 300 | merged | NA | NA |
| GSMUA_AchrUn_randomT02630_001_Phosphatidylinositol-4-phosphate_5-kinase_9:1485-1673 | 23.3856 | 329 |  | 167 | 44 |
| GSMUA_AchrUn_randomT04950_001_Probable_cellulose_synthase_A_catalytic_subunit_3_UDP-forming:1034-1380 | 33.1127 | 263 |  | 593 | 166 |
| GSMUA_AchrUn_randomT04950_001_Probable_cellulose_synthase_A_catalytic_subunit_3_UDP-forming:2340-2691 | 52.4951 | 407 | merged | NA | NA |
| GSMUA_AchrUn_randomT04950_001_Probable_cellulose_synthase_A_catalytic_subunit_3_UDP-forming:2691-3276 | 35.3233 | 293 | merged | NA | NA |
| GSMUA_AchrUn_randomT07140_001_expressed_protein:1350-1524 | 32.2208 | 309 |  | 167 | 37 |
| GSMUA_AchrUn_randomT09460_001_Probable_cellulose_synthase_A_catalytic_subunit_2_UDP-forming:1938-2289 | 68.3175 | 423 |  | 299 | 87 |
| GSMUA_AchrUn_randomT09650_001_Glucosamine--fructose-6-phosphate_aminotransferase_isomerizing_2:1282-1533 | 31.947 | 421 |  | 509 | 138 |
| GSMUA_AchrUn_randomT09650_001_Glucosamine--fructose-6-phosphate_aminotransferase_isomerizing_2:1648-1904 | 33.5262 | 364 | merged | NA | NA |
| GSMUA_AchrUn_randomT09650_001_Glucosamine--fructose-6-phosphate_aminotransferase_isomerizing_2:960-1282 | 26.2971 | 325 | merged | NA | NA |
| GSMUA_AchrUn_randomT09700_001_Clathrin_heavy_chain_1:2841-3327 | 65.6665 | 684 |  | 344 | 100 |
| GSMUA_AchrUn_randomT21380_001_auxin_efflux_carrier_component_putative_expressed:574-1179 | 27.9549 | 267 |  | 164 | 62 |
| GSMUA_AchrUn_randomT25710_001_Elongation_factor_1-alpha:159-574 | 28.8289 | 455 |  | 212 | 64 |
| GSMUA_AchrUn_randomT27420_001_Polyubiquitin_10:0-279 | 67.1779 | 316 |  | 245 | 78 |
| GSMUA_AchrUn_randomT27920_001_Charged_multivesicular_body_protein_1:0-387 | 59.6364 | 373 |  | 266 | 74 |
| whole plastid | 400 | 112193 | introns and intergenic spacers removed | 56202 | 8336 |
| accD | 180.955 | 1508 |  | 2333 | 426 |
| atpA | 327.729 | 1523 |  | 1535 | 226 |
| atpB | 287.432 | 1495 |  | 1517 | 192 |
| atpE | 207.245 | 408 |  | 407 | 58 |
| atpF | 715.442 | 1352 |  | 551 | 94 |
| atpH | 139.034 | 246 |  | 245 | 26 |
| atpI | 233.304 | 744 |  | 746 | 83 |
| ccsA | 172.912 | 985 |  | 1115 | 222 |
| cemA | 178.681 | 688 |  | 704 | 121 |
| clpP | 187.296 | 2107 |  | 650 | 185 |
| infA | 109.673 | 235 |  | 263 | 72 |
| matK | 685.711 | 1536 |  | 1613 | 337 |
| ndhA | 265.892 | 2147 |  | 1091 | 148 |
| ndhB | 452.896 | 2233 | skewed tree length | NA | NA |
| ndhC | 188.864 | 363 |  | 359 | 34 |
| ndhD | 866.049 | 1506 |  | 1526 | 290 |
| ndhE | 145.404 | 306 |  | 308 | 42 |
| ndhF | 186.075 | 2213 |  | 2618 | 505 |
| ndhG | 188.565 | 530 |  | 533 | 94 |
| ndhH | 2860.15 | 1182 |  | 1184 | 163 |
| ndhI | 2079.06 | 542 |  | 551 | 81 |
| ndhJ | 190.557 | 479 |  | 476 | 52 |
| ndhK | 248.12 | 773 |  | 887 | 107 |
| petA | 1411.07 | 963 |  | 959 | 116 |
| petB | 210.765 | 1463 |  | 650 | 65 |
| petD | 207.859 | 1234 |  | 479 | 60 |
| petG | 37.8546 | 110 |  | 110 | 9 |
| petL | 19.0491 | 90 | skewed tree length | NA | NA |
| petN | 16.9701 | 83 | skewed tree length | NA | NA |
| psaA | 2328.49 | 2253 |  | 2405 | 208 |
| psaB | 2411.99 | 2205 |  | 2207 | 201 |
| psaC | 137.404 | 246 |  | 242 | 40 |
| psaI | 31.4816 | 107 |  | 107 | 12 |
| psaJ | 48.4175 | 127 |  | 131 | 14 |
| psbA | 249.214 | 1062 |  | 1058 | 131 |
| psbB | 265.342 | 1527 | skewed tree length | NA | NA |
| psbC | 222.367 | 1422 |  | 1418 | 147 |
| psbD | 268.887 | 1062 |  | 1058 | 86 |
| psbE | 152.877 | 252 |  | 248 | 22 |
| psbF | 69.308 | 119 | skewed tree length | NA | NA |
| psbH | 104.955 | 221 |  | 218 | 24 |
| psbI | 27.1717 | 106 | skewed tree length | NA | NA |
| psbJ | 60.2015 | 122 |  | 125 | 14 |
| psbK | 78.0455 | 184 |  | 182 | 33 |
| psbL | 55.4941 | 116 | skewed tree length | NA | NA |
| psbM | 32.6341 | 101 |  | 101 | 9 |
| psbN | 62.5067 | 131 |  | 128 | 15 |
| psbT | 28.2031 | 101 |  | 110 | 8 |
| psbZ | 96.0475 | 188 |  | 185 | 15 |
| rbcL | 226.655 | 1458 |  | 1490 | 180 |
| rpl14 | 176.613 | 369 |  | 389 | 74 |
| rpl16 | 160.171 | 1317 |  | 407 | 87 |
| rpl2 | 155.415 | 390 |  | 827 | 35 |
| rpl20 | 1806.14 | 392 |  | 668 | 89 |
| rpl22 | 209.933 | 283 |  | 530 | 102 |
| rpl23 | 416.872 | 1487 | skewed tree length | NA | NA |
| rpl32 | 67.9864 | 171 |  | 242 | 39 |
| rpl33 | 99.5671 | 201 |  | 233 | 42 |
| rpl36 | 37.2246 | 112 | skewed tree length | NA | NA |
| rpoA | 204.112 | 1021 |  | 1052 | 196 |
| rpoB | 345.951 | 3229 |  | 3563 | 477 |
| rpoC1 | 239.031 | 2800 |  | 2168 | 308 |
| rpoC2 | 246.265 | 4140 |  | 4586 | 782 |
| rps11 | 181.792 | 416 |  | 452 | 121 |
| rps12 | 340.496 | 914 |  | 380 | 26 |
| rps14 | 158.51 | 303 |  | 302 | 58 |
| rps15 | 177.986 | 272 |  | 284 | 66 |
| rps16 | 173.363 | 1088 |  | 254 | 39 |
| rps18 | 145.327 | 319 |  | 431 | 50 |
| rps19 | 174.1 | 279 |  | 368 | 79 |
| rps2 | 210.657 | 711 |  | 737 | 153 |
| rps3 | 215.101 | 647 |  | 680 | 169 |
| rps4 | 201.868 | 609 |  | 659 | 95 |
| rps7 | 305.829 | 475 |  | 599 | 65 |
| rps8 | 143.959 | 397 |  | 407 | 73 |
| ycf1 | 260.843 | 5554 | alignment uncertainty | NA | NA |
| ycf15 | 265.772 | 495 |  | 509 | 28 |
| ycf2 | 419.459 | 6841 | skewed tree length | NA | NA |
| ycf3 | 229.438 | 2015 | skewed tree length | NA | NA |
| ycf4 | 177.162 | 535 |  | 584 | 116 |
| ycf68 | 597.076 | 385 | skewed tree length | NA | NA |
