# Supplementary figures and images for: Revisiting the Zingiberales: using multiplexed exon capture to resolve ancient and recent phylogenetic splits in a charismatic plant lineage
Source: PeerJ. 2016 Jan 21;4:e1584. doi: 10.7717/peerj.1584 (PMC4727956; doi:10.7717/peerj.1584)

**Histogram of average coverage per exon**

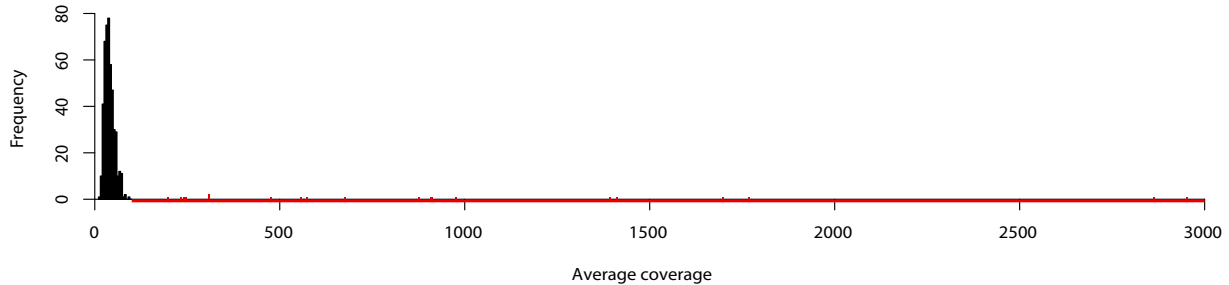

Supplement: Supplemental Information 4 — A red bar indicates the outlier area (coverage greater than 4 times the absolute median deviation). Frequencies for those exons that had coverage that fell within the outlier area are also highlighted in red. [file peerj-04-1584-s004.pdf]
